# Tribological Behavior and Attachment Force Regulation of Bioinspired Claw–Spines

**DOI:** 10.3390/biomimetics11080549

**Published:** 2026-08-03

**Authors:** Yanan Zhang, Xinlong Wu, Hongjian Wu, Xuan Wu, Feng Zhang, Baolin Jia, Xinping Li, Jing Pang

**Affiliations:** 1 College of Mechatronics Engineering, Henan University of Science and Technology, Luoyang 471003, China; zyn@haust.edu.cn (Y.Z.); wxl0014@stu.haust.edu.cn (X.W.); zf@stu.haust.edu.cn (F.Z.); blj@stu.haust.edu.cn (B.J.); 2 Institute of Advanced Manufacturing Technology, Hefei Institutes of Physical Science, Chinese Academy of Sciences, Changzhou 213164, China; 3 College of Agricultural Equipment Engineering, Henan University of Science and Technology, Luoyang 471003, China; aaalxp@126.com (X.L.); pangjing@haust.edu.cn (J.P.); 4 College of Mechanical & Electrical Engineering, Hohai University, Changzhou 213200, China

**Keywords:** bio-inspired claw–spine, tribological behavior, attachment mechanics, attachment force, wall-climbing robot

## Abstract

This study investigates the tribological behavior of the attachment force between bio-inspired claw–spines and rough surfaces and conducts theoretical and model-based analyses of the corresponding attachment mechanism. Based on critical interfacial tribological theory, a frictional mechanics model for a single claw–spine interacting with an arbitrary rough surface was developed. Furthermore, a stiffness-matrix-based mechanical model of bio-inspired claw–spine attachment to arbitrary surfaces was established, together with a contact interaction model between the claw–spine and the contact surface. The force distribution during the contact and attachment of the claw–spine to arbitrary surfaces was analyzed in detail. Criteria for determining stable claw–spine attachment were formulated, and the safe range of frame displacement variation under the corresponding conditions was identified. Based on these analytical results, a test platform for the bio-inspired claw–spine attachment structure was designed. In the experiments, the developed attachment-force measurement system was used to measure and analyze the frictional attachment forces generated by the claw–spine foot on different rough surfaces. After the claw–spine entered the stable attachment stage, the maximum claw–spine attachment forces measured on 60-grit, 80-grit, and 120-grit sandpaper surfaces were 0.62 N, 0.54 N, and 0.61 N, respectively. The corresponding maximum attachment forces measured on horizontal and vertical brick surfaces were 0.92 and 0.99 N, respectively. The results provide a basis for the mechanical analysis and force sensing of bio-inspired claw–spine attachment states and establish theoretical and experimental foundations for subsequent research on attachment-state recognition and motion control based on attachment-force feedback.

## 1. Introduction

Wall-climbing operations are widely required in fields such as construction, shipbuilding, quality inspection, and security. Manual wall-climbing operations pose significant safety risks, whereas wall-climbing robots can replace humans in performing such tasks under various environmental conditions, offering broad application prospects and substantial practical demand. However, conventional wall-climbing methods cannot fully satisfy the operating requirements of these specialized environments. For example, pneumatic adhesion systems cannot operate in vacuum environments [1,2], magnetic adhesion systems are restricted to ferromagnetic surfaces [3,4], and rail-guided wall-climbing systems require the prior installation of a complete track system [5,6]. Moreover, these attachment methods generally require robots to carry bulky devices, such as electromagnetic adhesion modules and vacuum suction units, which increase the overall size and mass of the robots and hinder their miniaturization. Consequently, such robots are unsuitable for tasks such as military reconnaissance and exploration in confined spaces. These limitations reduce the adaptability of wall-climbing robots to different surface conditions and restrict their practical applications [7,8].

In nature, many insects can move freely on walls and ceilings. However, the mechanisms underlying their attachment to surfaces remained incompletely understood for a long time [9,10,11]. In recent years, advances in scientific theory and observational methods have enabled extensive investigations into the biological attachment mechanisms between insect feet and attachment surfaces, revealing that insect attachment forces mainly arise from interfacial effects between the foot attachment structures and the substrate, including mechanical interlocking, friction, van der Waals forces, and fluid-mediated capillary and viscous forces [12,13,14]. Insect feet typically possess multiple structures involved in attachment, including pretarsal claws located at the distal ends of the feet, spine-like structures in the tarsal region, and various forms of adhesive pads or friction pads. The functions of these structures are closely related to the morphology and roughness of the contact surface. For example, insects such as *Serica orientalis* use their distal claws and tarsal spines to mechanically interlock with surface asperities and generate friction, thereby achieving stable attachment and locomotion on rough surfaces. Dai et al. investigated the frictional performance of the distal claws of the beetle *Pachnoda marginata* on surfaces with different roughness levels. The results showed that the relative scale between the claw tips and surface asperities is an important factor affecting claw attachment performance [15]. Song et al. investigated the combined effects of claws and adhesive pads on surface asperities of different scales and indicated that claw structures mainly adapt to rough surfaces through mechanical interlocking and frictional locking, whereas the synergistic action of claws and adhesive pads broadens the range of substrates to which the foot attachment system can adapt [16]. Winand et al. progressively removed the pretarsal claws of the stick insect *Sungaya inexpectata* and found that the absence of claws reduced its attachment capability on substrates with different roughness levels and affected the functions of other distal attachment structures [17]. By mimicking biological attachment mechanisms, researchers have developed bio-inspired wall-climbing robots equipped with bio-inspired structures, aiming to achieve wall climbing with low energy consumption and high adaptability, and some progress has been made in this field.

The first approach uses micro- and nanofabrication techniques to produce bioinspired dry adhesive materials. By mimicking the setae and terminal micro-/nanostructures on gecko feet, these materials generate reversible dry adhesion with climbing surfaces. They can be integrated into the feet, wheels, or tracked attachment mechanisms of wall-climbing robots, thereby enabling stable attachment and locomotion on walls [18,19,20]. The second approach employs advanced manufacturing technologies, including 3D printing, flexible-material forming, and smart-material actuation, to construct bioinspired attachment structures that mimic the flexibility of the tarsi and the functions of the terminal claws of beetles and other insects. These structures are applied to climbing on complex surfaces, such as mesh surfaces, inclined walls, and tree trunks [21,22,23]. Because bio-inspired dry-adhesion materials remain technically immature and are susceptible to wear and contamination, wall-climbing robots based on micro- and nanofabricated dry-adhesion structures are still largely confined to laboratory research. In contrast, bio-inspired claw–spine attachment structures exhibit relatively high reliability in practical environments. Therefore, wall-climbing robots based on bio-inspired claw–spine attachment are expected to find near-term applications in disaster rescue, wall-surface reconnaissance, planetary exploration, and other fields. For example, the National Aeronautics and Space Administration has developed planetary exploration robots that use claw–spine attachment and has tested them in simulated environments [24,25].

Current research on bio-inspired claw–spine wall-climbing robots has primarily focused on the design of claw–spine attachment structures and robot system integration. For example, robot attachment performance has been improved by optimizing claw–spine structures and gait design. However, research on attachment-force sensing and control for robotic claw–spines remains largely unexplored. This capability is essential for bio-inspired claw–spine wall-climbing robots to accurately identify their attachment states and achieve autonomous and stable motion control. Nadan et al. proposed the lightweight free-climbing robot LORIS in 2024, which employs microspine grippers with independently compliant elements to adapt to brick walls and irregular rock surfaces and uses an optimization-based method to distribute loads among different grippers, thereby reducing the risks of local overloading and accidental detachment [26]. Xiang et al. investigated the scale-dependent effects of bio-inspired claw–spine arrays on soft and hard substrates and, in a subsequent study, further analyzed the influence of claw–spine orientation on the surface interlocking process, demonstrating that increasing the number of claw–spines does not necessarily produce a proportional increase in load-bearing capacity and that both claw–spine arrangement and loading direction are important parameters governing effective interlocking [27,28]. Zhu et al. pointed out that surface adaptability, sensing capability, and control strategies are key directions for improving the applicability of wall-climbing robots in complex environments [29]. Kong et al. further reviewed the progression from biological attachment mechanisms to engineering biomimetic implementations, covering hook-and-claw attachment, dry adhesion, wet adhesion, and the corresponding control methods, and identified the integration of sensing and adaptive control as an important development trend for bio-inspired wall-climbing robots [30]. Therefore, developing attachment-force sensing methods for bio-inspired claw–spine systems by integrating biomimetics, mechanics, sensor technology, control theory, and other disciplines can effectively improve the performance of key components and the overall robot, thereby providing a technological foundation for the development of bio-inspired wall-climbing robots.

In summary, current research on bio-inspired claw–spine wall-climbing robots, both domestically and internationally, has mainly focused on attachment mechanism analysis, claw–spine structure design, and robot system integration. Regarding the analysis of attachment forces between claw–spine foot attachment structures and contact surfaces, previous studies have optimized claw–spine structural parameters to enable passive adaptation to the surface morphology, thereby enhancing mechanical interlocking between the claw–spines and the wall. However, the foot motion of existing bio-inspired claw–spine wall-climbing robots is mostly controlled using open-loop strategies, and real-time sensor-based methods for attachment-force sensing remain lacking. Moreover, the relationships among the tribological behavior of the claw–spine attachment interface, structural deformation, and attachment-force response have not yet been systematically investigated. Consequently, robots operating on complex wall surfaces have difficulty obtaining their attachment states in a timely manner, which further limits subsequent research on force-feedback-based state recognition and autonomous adjustment.

Because attachment force is an important parameter reflecting the claw–spine–wall contact state and attachment stability, investigating the frictional mechanical behavior of the claw–spine attachment interface and establishing a reliable attachment-force detection method are of great significance for improving the environmental adaptability and motion stability of bio-inspired claw–spine wall-climbing robots.

To address these issues, this study investigates the frictional behavior of the bio-inspired claw–spine attachment interface, analyzes the mechanical response throughout the attachment process from initial contact and stable attachment to detachment, and develops a bio-inspired claw–spine attachment-force measurement system capable of real-time force detection during attachment. The results provide a theoretical and experimental basis for subsequent attachment-state recognition and autonomous control based on force feedback. The main research contents are as follows: (1) A frictional contact mechanics model between a claw–spine and a curved surface and a bilateral opposed cooperative claw–spine attachment model were established, enabling the derivation of the attachment force expression, stable attachment criterion, and safe displacement range. (2) A bilateral rigid–flexible hybrid claw–spine opposed cooperative attachment structure was designed, and an attachment-force measurement system capable of real-time measurement was developed. (3) Quasi-static attachment tests were performed on different rough surfaces, and the theoretical model and measurement system were validated by comparison with the commercial LSM300 sensor.

## 2. Bio-Inspired Mechanism of Flexible Claw–Spines

### 2.1. Design of the Bio-Inspired Flexible Claw–Spine Structure

When insects crawl on rough surfaces, the normal claw–spine attachment force at the spine tip is perpendicular and inward to the contact surface, which counteracts the torque caused by gravity and prevents insects from sliding down the rough surface. The tangential claw–spine attachment force is parallel to the contact surface and provides the force required for forward locomotion while counteracting the insect’s own gravity. Because a positive correlation exists between the normal load-bearing capacity and the tangential force within a certain range, the flexible opposed cooperative claw–spine attachment structure formed by the claws and spines at the distal end of insect feet can provide sufficient tangential force at multiple angles, thereby increasing the normal load and improving the attachment stability of insects on rough surfaces.

**Figure 1:**
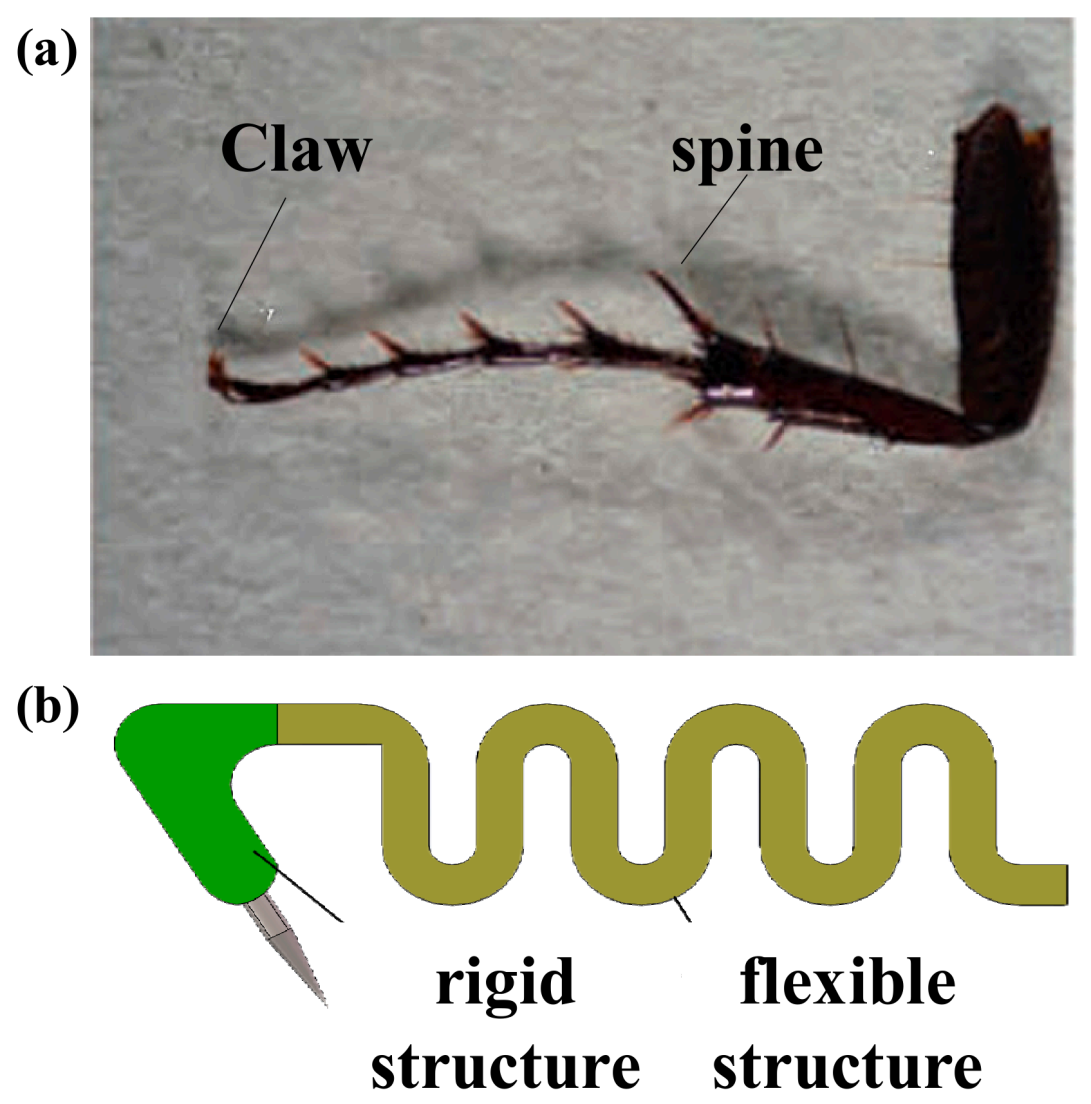
Development of the biomimetic claw–spine model. (**a**) Adult *Serica orientalis* [31] and Tarsal claw–spine structure [32]. Reprinted with permission from Ref. [32]. Copyright 2015, Yanwei Liu. (**b**) Design model of a single bio-inspired claw–spine.

Adult *Serica orientalis* inhabits tree branches and leaf surfaces and is a typical insect capable of climbing on rough surfaces [31]. Figure 1a shows one of its feet, in which the tarsus, composed of several tarsomeres and connected to the tibia, bears multiple tarsal spines, while a pretarsal claw is located at the distal end [32]. *Serica orientalis* uses the distal claw and tarsal spines of its foot to interact frictionally with rough wall surfaces. Through mechanical interlocking, the claw and tarsal spines form a flexible, opposing, and coordinated attachment structure, enabling the insect to attach to rough surfaces [33,34,35].

The distal claw and tarsal spines of *Serica orientalis* were selected as morphological references for the bio-inspired design. Rather than geometrically reproducing the insect foot structure at the same scale, the design extracts its functional characteristics, including the coordinated attachment of the claw and tarsal spines and their mechanical interlocking with rough surfaces. On this basis, a design model of a single flexible bio-inspired claw–spine was established, as shown in Figure 1b. Multiple bio-inspired claw–spines were then arranged in arrays on both sides of the mechanism to form a bio-inspired claw–spine wall-climbing mechanism capable of opposing and coordinated attachment.

Since the *Serica orientalis* is a typical insect capable of climbing rough surfaces, its tarsal segments lack any adhesive pads. The spiny structures interact with microscopic surface protrusions to generate frictional contact forces. Therefore, it can be considered that the beetle relies solely on the attachment force generated by the flexible opposing synergistic claw–spine attachment structure formed by the terminal claws and spines for climbing.

During the design of the claw–spine structure, coupling between the tangential and normal stiffnesses may prevent the claw–spine tip from disengaging smoothly from the contact surface while it is in the attached state. To address this issue, an endoskeleton-constrained flexible claw–spine structure can be adopted to independently regulate the tangential and normal stiffnesses.

The individual flexible claw–spine foot element was designed according to a rigid–flexible integration strategy. On the one hand, the foot element must possess sufficient resistance to fracture under loading. On the other hand, to satisfy both miniaturization and high-load requirements, it must retain sufficient extensibility despite its relatively short initial length. Therefore, the individual compliant claw–spine foot element adopts an S-shaped configuration and is fabricated from two materials with different hardness levels. The harder front section serves as the rigid component for mechanical analysis, whereas the softer rear section provides high flexibility and elastic recovery, preventing load-induced failure and accommodating large deformation. This design facilitates reliable attachment and disengagement of the claw–spine while improving its mechanical performance and adaptability. The overall structure is shown in Figure 1b.

A detailed investigation of the attachment process of claw–spines on rough surfaces, together with an analysis of changes in the contact forces between the claw–spines and the rough surface, provides an important basis for designing flexible claw–spine structures with excellent attachment performance. Observations show that the attachment process between a claw–spine and a rough surface involves several stages, including initial contact, engagement, stable attachment, and possible sliding or detachment. Because both the attachment process and the surface morphology are complex, the analysis is simplified by first investigating the variation in contact force between a single claw–spine and an individual surface asperity, and then examining the attachment force between a claw–spine array and a rough surface, thereby providing a basis for the development of a more efficient attachment system.

### 2.2. Investigation of the Frictional Attachment Force at the Interface of a Single Claw–Spine

**Figure 2:**
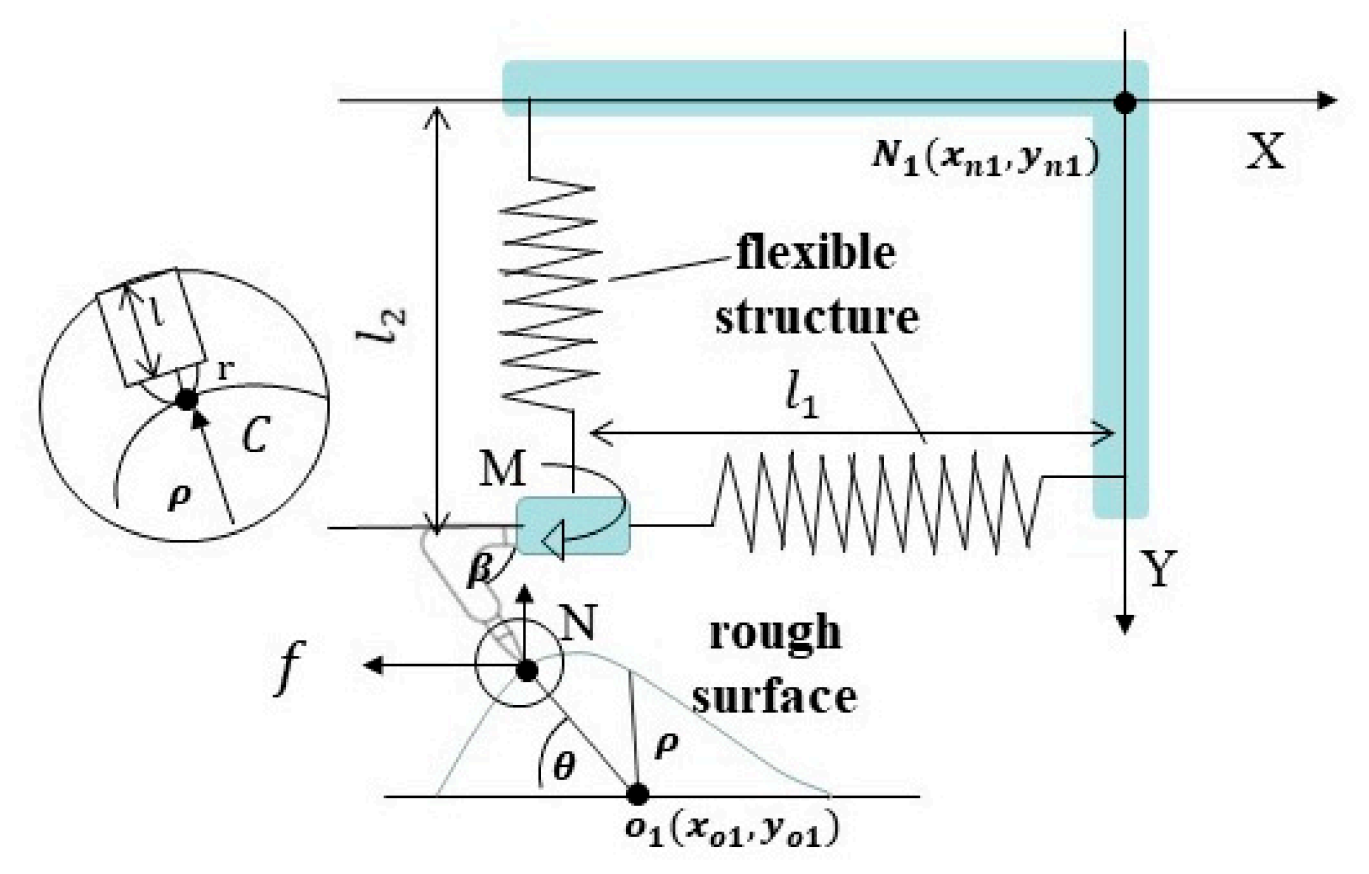
Frictional mechanics model of contact between a single claw–spine and a surface asperity.

In nature, the spine-like structures on insect tarsi generate attachment forces through this mechanical mechanism. When an insect climbs on a rough surface, the claw–spines at the distal end of the tarsal structure mechanically interlock with surface asperities. When the asperities on the contact surface are randomly distributed, the flexible tarsal links can provide cushioning and regulate the attachment force, thereby enabling stable attachment. During loading, the claw–spine is subjected to forces in both the anterior–posterior and vertical directions. Based on this mechanism, the distal end of the tibia was simplified as a revolute joint, and a mechanical model of the claw–spine in contact with the surface was established. Accordingly, a mechanical analysis model describing the initial contact between a single claw–spine structure and an individual surface asperity was developed, as shown in Figure 2.

Figure 2 presents a schematic diagram for analyzing the attachment forces acting on a single claw–spine element in contact with a surface. The claw–spine is connected to the frame through two spring-like compliant structures in the tangential and normal directions, and the contact forces generated between the claw–spine and the rough surface are defined as the claw–spine attachment forces. Here, f denotes the tangential attachment force, whose direction coincides with the tangent to the surface asperity at the claw–spine contact point and is parallel to the local contact plane. This force facilitates attachment to the contact surface and reduces the likelihood of claw–spine sliding. N denotes the normal attachment force, which is perpendicular to the tangent of the surface asperity at the contact point. This force contributes to maintaining contact with the surface and, to a certain extent, reduces the likelihood of detachment. M denotes the torque induced by the self-weight of the claw–spine and by variations in the contact angle between the claw–spine and the surface asperity. Therefore, f, N, and M were selected as the principal variables for establishing the mechanical model of a single claw–spine during its initial contact with an arbitrary surface.

$l_{1}$ is the original length of the tangential compliant structure of the claw–spine element, and $l_{2}$ is the original length of its normal compliant structure. C represents the center point of the claw–spine footpad frame, β is the attachment angle at the connection between the claw–spine and the footpad frame, and θ is the contact angle between the claw–spine tip and the asperity on the attachment surface.

**Figure 3:**
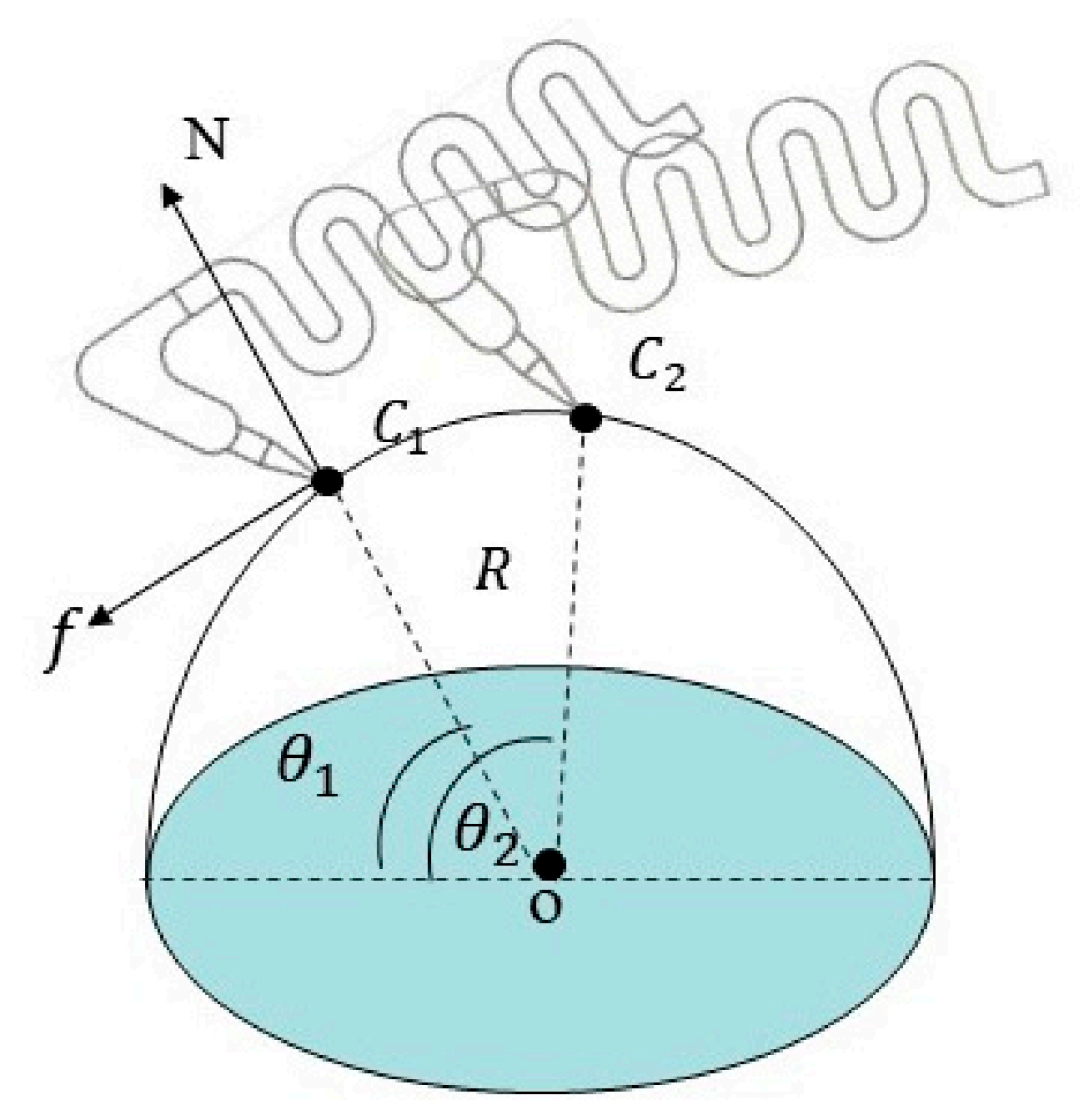
Schematic diagram of a single claw–spine sliding over surface asperities during contact.

The rough surface asperity involved in attachment was represented by an arbitrary curve equation $y\;=\;f\left(x\right)$, where both x and f represent length quantities. The first derivative $f^{\prime }\left(x\right)$ is a dimensionless quantity, while the second derivative $f^{\prime \prime }\left(x\right)$ has the dimension of reciprocal length. A coordinate system was established as shown in Figure 3. For any point on this curve, the contact position between the claw–spine and the attachment surface can be determined. The claw–spine was simplified as a cylindrical–hemispherical structure, consisting of a cylinder with a height of l and a hemispherical tip with a radius of r. During the initial contact stage, the contact point between the claw–spine and the surface asperity was defined as $C_{1}$. The center of curvature at $C_{1}$ was defined as $O_{1}$, with a curvature radius of ρ. The line connecting $C_{1}$ and $O_{1}$ forms an angle θ with the horizontal direction, and the initial inclination angle of the claw–spine relative to the x-axis is defined as β.

The curvature radius ρ can be expressed as:

(1) $$\rho \;=\;\frac{{(1\;+\;{f^{\prime }\left(x\right)}^{2})}^{\frac{3}{2}}}{\left|f^{\prime \prime }\left(x\right)\right|}$$

For the plane curve $y\;=\;f\left(x\right)$, the direction of curvature at the contact point is determined by the signed curvature. The coordinates of the center of curvature $O_{1}$ are given as:

(2) $$\left\{\begin{array}{l} x_{o1}\;=\;x\;-\;f^{\prime }(x)\frac{1\;+\;{f^{\prime }(x)}^{2}}{f^{\prime \prime }(x)}\\ y_{o1}\;=\;f\left(x\right)\;+\;\frac{1\;+\;{f^{\prime }(x)}^{2}}{f^{\prime \prime }(x)} \end{array}\right.$$

In these equations, the sign of $f^{\prime \prime }\left(x\right)$ determines the position of the center of curvature relative to the normal direction of the curve. When the concavity of the curve changes, the sign of $f^{\prime \prime }\left(x\right)$ changes accordingly, resulting in a corresponding change in the position of the center of curvature. Let the coordinates of the reference point of the claw–spine frame be $N_{1}(x_{n1},y_{n1})$. Under the initial contact condition, the center of curvature is $O(x_{O1},y_{O1})$, and the following relationship is obtained:

(3) $$\left\{\begin{array}{l} x_{o1}\;={\;x}_{n1}-l_{1}\;+\;l\cos\beta \;+\;(\rho \;+\;r)\cos\theta \\ y_{o1}\;=\;y_{n1}-l_{2}-l\sin\beta -(\rho \;+\;r)\sin\theta \end{array}\right.$$

When the claw–spine moves with the claw–spine frame and begins to engage and drag along the attachment surface until reaching a stable mechanical interlocking position, the coordinates of the center of curvature of the contact asperity and the claw–spine frame center both change. Let the center of curvature after attachment dragging be defined as ${O_{1}}^{\prime }\left({x_{O1}}^{\prime },{y_{O1}}^{\prime }\right)$, and the frame center coordinate be ${N_{1}}^{\prime }({x_{n1}}^{\prime },{y_{n1}}^{\prime })$. At this stage, the coordinates of the center of curvature become:

(4) $$\left\{\begin{array}{l} {x_{O1}}^{\prime }\;=\;{x_{n1}}^{\prime }-{l_{1}}^{\prime }\;+\;l\cos\beta^{\prime }+\;(\rho \;+\;r)\cos\theta^{\prime }\\ {y_{O1}}^{\prime }=\;{y_{n1}}^{\prime }-{l_{2}}^{\prime }-l\sin\beta^{\prime }-(\rho \;+\;r)\sin\theta^{\prime } \end{array}\;\right.$$

where $\beta^{\prime }$ represents the angle between the claw–spine and the x-axis after contact, which corresponds to the current contact angle after bending deformation. Let the deformation of the tangential compliant claw–spine structure in the x-direction be defined as $\Delta l_{1\;}=\;{l_{1}}^{\prime }-l_{1}$, the deformation of the normal compliant claw–spine structure in the y-direction be defined as $\Delta l_{2}\;=\;{l_{2}}^{\prime }-l_{2}$, and the angular deformation be $∆\beta \;=\;\beta^{\prime }-\beta$. Then, the following relationship can be obtained:

(5) $$\left\{\begin{array}{c} \Delta l_{1}\;={{\;x}_{n1}}^{\prime }-x_{n1}\;+{\;x}_{o1}-{x_{O1}}^{\prime }-l\left(\cos\beta -\cos\beta^{\prime }\right)\\ -\;(\rho \;+\;r)(\cos\theta -\cos\theta^{\prime })\\ \Delta l_{2}\;=\;{y_{n1}}^{\prime }-y_{n1}\;+\;y_{O1}-{y_{O1}}^{\prime }\;+\;l\left(\sin\beta -\sin\beta^{\prime }\right)\\ +\;\left(\rho \;+\;r\right)(\sin\beta -\sin\beta^{\prime }) \end{array}\right.$$

where Δβ represents the variation in the rotation angle, and the stiffness matrix of the claw–spine is defined as K. Then, F = KΔr. Where $F\;=\;{\left(f_{x},{\;f}_{y},\;M\right)}^{\prime }$ represents the tangential force, normal force, and torque acting at the tip of the claw–spine, respectively. $\Delta r\;{=\;(\Delta x,\Delta y,\Delta \beta )}^{\prime }$; and $M\;=\;\mathrm{fl}\sin\beta^{\prime }+\;\mathrm{Nl}\cos\beta^{\prime }$. Thus

(6) $$\left(\begin{array}{c} f_{x}\\ f_{y}\\ f_{x}l\sin\beta^{\prime }+{\;f}_{y}l\cos\beta^{\prime } \end{array}\right)=\left(\begin{array}{lll} k_{l_{1}l_{1}} & k_{l_{1}l_{2}} & k_{l_{1}\beta }\\ k_{l_{2}l_{1}} & k_{l_{2}l_{2}} & k_{l_{2}\beta }\\ k_{\beta l_{1}} & k_{\beta l_{2}} & k_{\beta \beta } \end{array}\right)\left(\begin{array}{l} \Delta l_{1}\\ \Delta l_{2}\\ ∆\beta \end{array}\right)$$

The tangential force of the claw–spine can be expressed as:

(7) $$f_{x}\;=\;k_{l_{1}l_{1}}\Delta l_{1}\;+\;k_{l_{1}l_{2}}\;\Delta l_{2}\;+\;k_{l_{1}\beta }∆\beta$$

The normal force of the claw–spine can be expressed as:

(8) $$f_{y}\;=\;k_{l_{2}l_{1}}\Delta l_{1}\;+\;k_{l_{2}l_{2}}\;\Delta l_{2}\;+\;k_{l_{2}\beta }∆\beta$$

The torque of the claw–spine can be expressed as:

(9) $$M\;={\;f}_{x}l\sin\beta^{\prime }\;+{\;f}_{y}l\cos\beta^{\prime }\;={\;k}_{\beta l_{1}}\Delta l_{1\;}+{\;k}_{\beta l_{2}}\;\Delta l_{2}\;+\;k_{\beta \beta }∆\beta$$

where the multidimensional forces and deformations experienced by the compliant claw–spine structure are coupled with each other. Therefore, its stiffness matrix can generally be expressed as:

(10) $$K=\left(\begin{array}{ccc} k_{l_{1}l_{1}} & k_{l_{1}l_{2}} & k_{l_{1}\beta }\\ k_{l_{2}l_{1}} & k_{l_{2}l_{2}} & k_{l_{2}\beta }\\ k_{\beta l_{1}} & k_{\beta l_{2}} & k_{\beta \beta } \end{array}\right)$$

where $k_{l_{1}l_{1}}$, $k_{l_{1}l_{2}}$, $k_{l_{2}l_{1}}$, and $k_{l_{2}l_{2}}$ represent the elastic support provided by the compliant structure to the claw–spine in the x and y directions. $k_{\beta l_{1}}$, $k_{\beta l_{2}}$, and $k_{\beta \beta }$ represent the torque -generating capability of the flexible claw–spine structure, while $k_{l_{1}\beta }$ and $k_{l_{2}\beta }$ describe the relationship between the deformations in the x and y directions and the rotational torque. In the stiffness matrix, $k_{l_{1}l_{1}}$ should be maintained at an appropriate level to enable adaptation to the average spacing of surface asperities while sustaining relatively large loads. $k_{l_{2}l_{2}}$ should be relatively small to allow millimeter-scale adaptation to surface geometric variations. In addition, $k_{l_{1}l_{2}}\;<\;0$ enables the remaining claw–spines to continue moving toward the wall after one claw–spine has anchored onto a surface asperity during the attachment process. This characteristic is a key parameter in the design of bio-inspired flexible claw–spine attachment structures.

When the claw–spine comes into contact with the climbing surface, it is necessary to determine whether effective attachment has been achieved. This provides a basis for establishing the control criteria for stable claw–spine attachment in subsequent studies. Figure 3 illustrates the variation in parameters of the claw–spine after dragging on the contact surface. Here, f represents the friction force acting on the claw–spine at the contact point, and N represents the normal force applied to the claw–spine at the contact point. When the compliant structure of the claw–spine element undergoes dragging along the contact surface, the contact angle between the claw–spine and the surface asperity changes from $\theta_{1}$ to $\theta_{2}$. O represents the center of curvature of the contact point on the attachment surface.$\;C_{1}$ denotes the initial contact point between the claw–spine and the climbing surface before dragging, with a contact angle of $\theta_{1}$, while $C_{2}$ denotes the contact point after dragging, with a contact angle of $\theta_{2}$.

The criterion for stable attachment of a claw–spine can be determined based on Coulomb’s law. If the claw–spine maintains stable attachment with the contact surface, the following conditions must be satisfied: the normal force exerted on the claw–spine should be non-negative, and the friction force should be lower than the maximum static friction force between the claw–spine and the contact surface. Therefore,

(11) $$\left\{\begin{array}{c} N\;>\;0\\ |f|\;\leq \;\mu N\;\\ N=f_{x}\sin\theta +f_{y}\cos\theta \\ f=f_{x}\cos\theta -f_{y}\sin\theta \end{array}\right.$$

where μ represents the coefficient of friction of the contact surface.

When the claw–spine tip forms stable contact with a rough-surface asperity, and no obvious relative sliding occurs at the contact interface over a small motion increment, the instantaneous contact can be approximated as a no-slip rolling state [33,36]. Accordingly, the instantaneous kinematic relationship between the claw–spine tip and the surface asperity can be expressed as follows:

(12) ρ∆θ = rΔβ

According to the tribological equation, the following relationship can be obtained:

(13) $$\left\{\begin{array}{c} f_{x}\tan\theta -f_{y}\;>\;0\\ 0\;<\;\frac{f_{x}\tan\theta -f_{y}}{f_{x}\;+\;f_{y}\tan\theta }\;\leq \;\mu \end{array}\right.$$

Let

$$\delta =f_{x}\tan\theta -f_{y},\;\varepsilon =\frac{f_{x}\tan\theta -f_{y}}{f_{x}+f_{y}\tan\theta }$$

For the bio-inspired claw–spine to achieve stable attachment on an arbitrary rough surface, the following conditions must be satisfied:

(14) $$\left\{\begin{array}{l} \delta \;>\;0\\ \varepsilon \;\leq \;\mu \end{array}\right.$$

Based on this criterion, the structural parameters of the bio-inspired claw–spine can be designed to ensure stable attachment on rough surfaces. When the spine tip of the claw–spine contacts the contact surface, the claw–spine applies normal pressure and generates stable tangential friction on the contact surface. According to the above model, the variation in contact force caused by the displacement of the frame can be calculated. Thus, the displacement range of the frame when the claw–spine maintains stable attachment and the maximum deformation threshold of the compliant claw–spine structure under the critical attachment condition can be obtained, effectively preventing attachment failure and structural damage of the claw–spine.

### 2.3. Analysis of the Frictional Attachment Characteristics of a Single Claw–Spine

**Figure 4:**
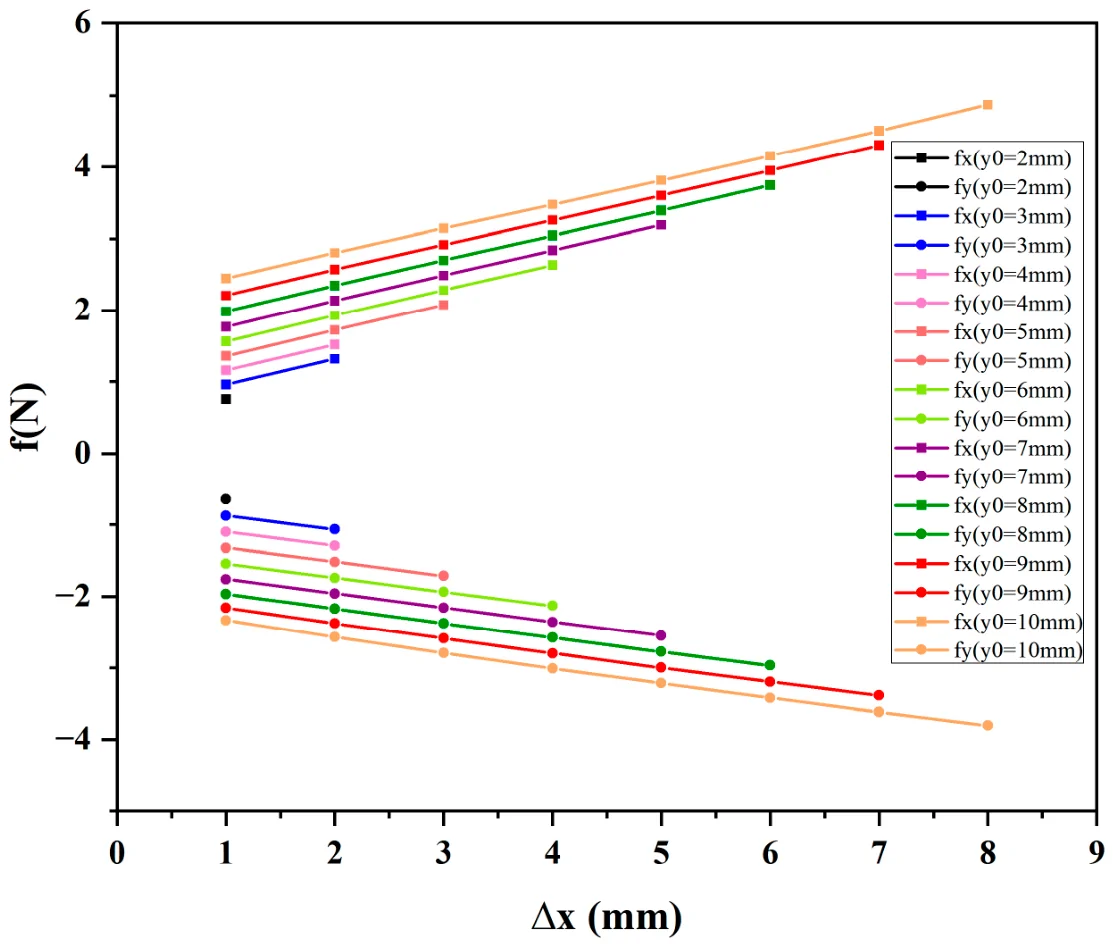
Relationship between tangential and normal forces of the bio-inspired claw–spine and deformation of the flexible structure.

The motion of the bio-inspired claw–spine is driven by the active pulling of the rigid–flexible hybrid structural frame of the claw–spine sheet, which subsequently drives the flexible connecting components and the terminal claw–spine structure, ultimately forming a dynamic deformation–force coupling effect at the contact interface. According to the contact mechanics model of a single claw–spine contacting a surface asperity established in Figure 4, the claw–spine attachment force is influenced by multiple factors, including the horizontal displacement of the frame (∆$l_{1}$), vertical displacement of the frame (∆$l_{2}$), rotation angle of the compliant frame structure (Δβ), contact point C (x, y) between the claw–spine and the contact surface, initial angle between the claw–spine and the frame β, and contact state angle between the claw–spine and the contact surface θ. These factors collectively affect the tangential force $f_{x}$, normal force $f_{y}$, and torque M acting on the claw–spine at the contact surface.

The flexible structural substrate of the claw–spine is considered a fixed invariant. Different groups of loads δ$\;\left(∆x,\;∆y,\;∆\alpha \right)$, are applied to the tip of the claw–spine structure. The mechanical analysis module in the software is then used to obtain the force response at the end of the claw–spine structure, which is expressed as $F\;=\;{\left(F_{x},{\;F}_{y},\;M\right)}^{\prime }$. In the finite element model, the contact forces in different directions can be obtained by applying prescribed displacements in corresponding directions. For example, by applying the displacement vectors (1 mm, 0, 0)′, (0, 1 mm, 0)′, (0, 0, 1 degree)′, the stiffness matrix K can be determined, such that $K\left(∆x,\;∆y,\;∆\alpha \right)\;=\;\left(F_{x},\;F_{y},\;M\right)$. For example, when the input displacement vector is (1 mm, 0, 0)′, the finite element software outputs the force response (${F_{t1},\;F}_{n1}{,\;T}_{1}$), from which we can obtain ($k_{\mathrm{xx}}$,$\;k_{\mathrm{yx}}$,$\;k_{\alpha x}$) = ($F_{t1}$,$\;F_{n1},\;T_{1}$)/1 mm. It should be noted that when obtaining another set of stiffness coefficients in the stiffness matrix, such as ($k_{x\alpha }$,$\;k_{y\alpha }$,$\;k_{\alpha \alpha }$), the displacement vector (0, 0, 1 degree)′ is applied to the tip of the claw–spine to simulate contact, where T represents the measured contact reaction force near the tip of the claw–spine, and K represents the stiffness matrix of the claw–spine structure to be determined. According to the above calculation process, the stiffness matrix of the finite element model can be obtained as follows:

(15) $$\left(\begin{array}{c} F_{x}\\ F_{y}\\ M \end{array}\right)\;=\;\left(\begin{array}{l} \frac{\partial F_{x}}{\partial x}\\ \frac{\partial F_{y}}{\partial x}\\ \frac{\partial T}{\partial x} \end{array}\begin{array}{l} \;\frac{\partial F_{x}}{\partial y}\;\\ \frac{\partial F_{y}}{\partial y}\\ \frac{\partial T}{\partial y} \end{array}\begin{array}{l} \frac{\partial F_{x}}{\partial \alpha }\\ \frac{\partial F_{y}}{\partial \alpha }\\ \frac{\partial T}{\partial \alpha } \end{array}\right)\left(\begin{array}{l} dx\\ dy\\ d\alpha \end{array}\right)$$

(16) $$K=\left(\begin{array}{lll} \frac{\partial F_{t}}{\partial x} & \frac{\partial F_{t}}{\partial y} & \frac{\partial F_{t}}{\partial \alpha }\\ \frac{\partial F_{n}}{\partial x} & \frac{\partial F_{n}}{\partial y} & \frac{\partial F_{n}}{\partial \alpha }\\ \frac{\partial T}{\partial x} & \frac{\partial T}{\partial y} & \frac{\partial T}{\partial \alpha } \end{array}\right)=\left(\begin{array}{lll} k_{\mathrm{xx}} & k_{\mathrm{xy}} & k_{x\alpha }\\ k_{\mathrm{yx}} & k_{\mathrm{yy}} & k_{y\alpha }\\ k_{\alpha x} & k_{\alpha y} & k_{\alpha \alpha } \end{array}\right)$$

The calculated stiffness matrix is

(17) $$K=\left(\begin{array}{ccc} 851.4 & 88.5 & -3.0639\\ 80.4 & 386.7 & -3.2396\\ -2.9828 & -3.3419 & 0.0406181 \end{array}\right)$$

The units of the stiffness matrix are

$$\left(\begin{array}{ccc} N/m & N/m & N/\mathrm{rad}\\ N/m & N/m & N/\mathrm{rad}\\ N/\mathrm{rad} & N/\mathrm{rad} & \mathrm{Nm}/\mathrm{rad} \end{array}\right)$$

Thus, the stiffness matrix of the claw–spine structure was obtained, enabling the structure to possess sufficient structural stability and facilitating the control of the claw–spine attachment force, thereby improving attachment stability. Based on the above analysis and finite element simulation of the attachment mechanics of a single compliant claw–spine, the safe loading displacement ranges of the frame in the x and y directions (i.e., the tangential and normal directions) under varying external frame loads can be determined. These displacement ranges ensure that the claw–spine maintains stable attachment when the external load increases. This approach conforms to the rigid–flexible hybrid design concept and enables passive adaptation to rough wall surfaces with different roughness characteristics.

A calculation program was developed in MATLAB R2024a. During the attachment of the bio-inspired claw–spine on a rough surface, the friction coefficient of the rough contact surface was set as 0.8. The attachment angle between the claw–spine and the footpad frame was set as β = 45°, and the initial contact angle between the claw–spine tip and the surface asperity was set as θ = 6°. The curvature radius of the wall asperity peak was 269 μm, the radius of the claw–spine tip was 20 μm, the claw–spine length was 3 mm, the tangential compliant structure length of the claw–spine was 20 mm, and the normal compliant structure length was 15 mm. Under the conditions of fixed initial contact angle, contact point position, and normal dragging displacement, the tangential dragging displacement was gradually increased. Taking the tangential displacement (∆$x\;=\;{l^{\prime }}_{1}-l_{1}$) as the independent variable, the initial horizontal coordinate of the contact point was set as $x_{c}\;=\;1\;\mathrm{mm}$. The normal pre-compression displacement of the compliant claw–spine was maintained at a constant value, increasing from 1 mm to 10 mm, while the tangential deformation of the compliant claw–spine structure was simultaneously increased from 1 mm to 10 mm with an increment of 1 mm. Based on the contact friction model of the claw–spine on rough surfaces (Equations (1)–(10)) and the stable attachment criterion (Equation (14)), the relationship between the tangential/normal claw–spine attachment force and tangential displacement of a single claw–spine was investigated.

By changing the tangential and normal loading displacements of the compliant structure of the bio-inspired claw–spine, different attachment states can be obtained. According to the stable attachment criterion of the bio-inspired claw–spine on arbitrary rough surfaces, the loading ranges of the normal and tangential displacements of the compliant structure corresponding to the attachment safety region of the claw–spine can be determined. Meanwhile, the critical attachment force of the claw–spine at the moment of detachment from the contact surface and the corresponding displacements in the x- and y-directions can be obtained. The normal force corresponding to the maximum tangential force can also be calculated. This calculation method can simulate the attachment process of the claw–spine on a known contact surface. The direction of the tangential force of the claw–spine is defined as the same as the positive x-axis direction, namely parallel to and upward along the contact surface, with a positive value. The direction of the normal force is defined as the same as the y-axis direction, namely parallel to and downward along the contact surface, with a negative value. When the pre-compression displacement of the compliant claw–spine structure is 1 mm, and the calculated results under other normal and tangential displacement variables do not satisfy the stable attachment criterion (10), the corresponding results are not displayed in the calculation plots.

Figure 4 shows the relationship between the tangential and normal claw–spine attachment forces and the deformation of the compliant structure. When the normal pre-compression displacement of the compliant structure remains constant, both the tangential and normal claw–spine attachment forces increase with increasing tangential loading displacement of the frame. The normal force is negative, indicating that its direction is opposite to the defined positive direction, i.e., vertically downward and perpendicular to the contact surface, which prevents the claw–spine from detaching from the contact surface. As the normal pre-compression displacement of the compliant structure of the bio-inspired claw–spine increases, the adjustable range of tangential deformation of the compliant structure becomes larger. Each group of experimental data exhibits a corresponding truncation point in the trend curve. The appearance of the truncation point indicates that the claw–spine has detached from the contact surface and no longer generates contact force, meaning that the stable attachment criterion (14) is no longer satisfied. The frame dragging displacement corresponding to this truncation point represents the maximum external load that the claw–spine can withstand, and the corresponding contact force represents the maximum contact force generated by the claw–spine under this condition.

**Figure 5:**
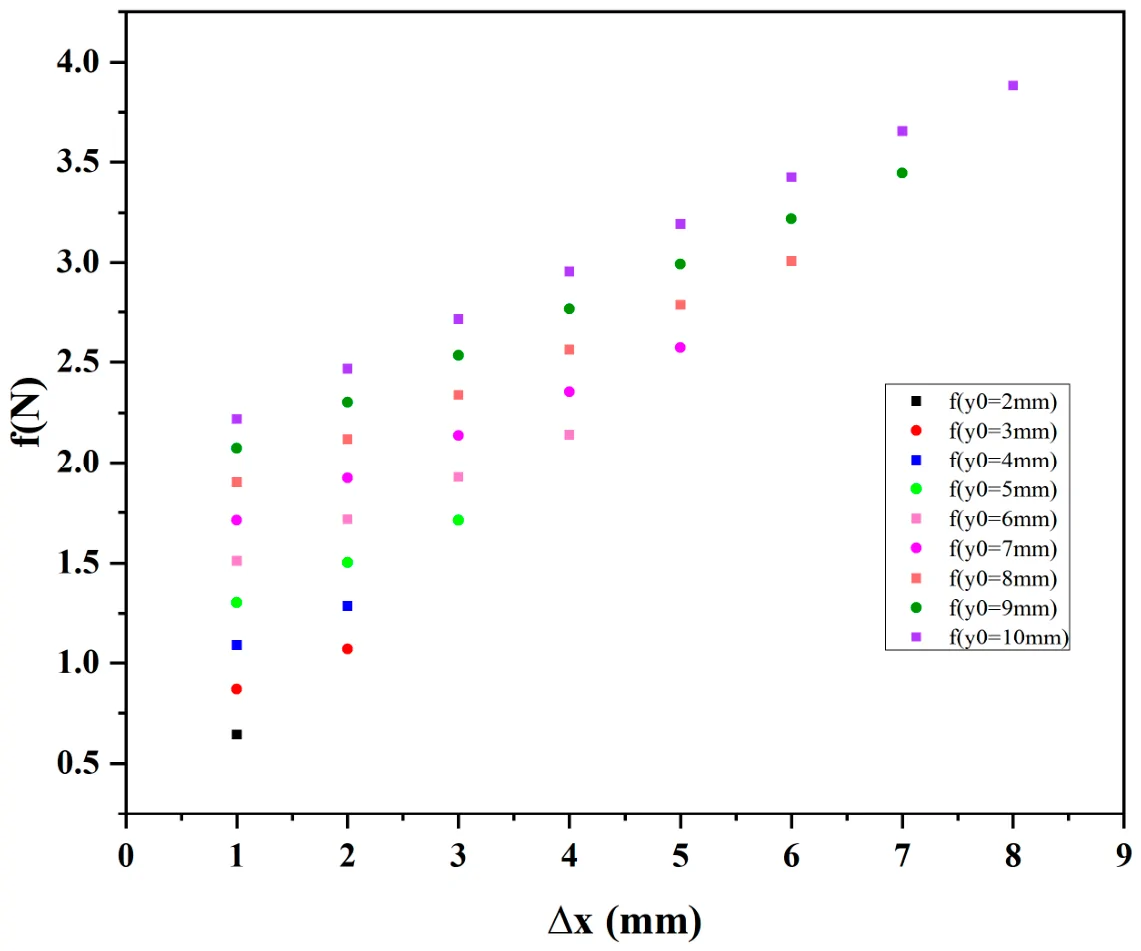
Relationship between friction force at the attachment surface of the bio-inspired claw–spine and deformation of the flexible structure.

Figure 5 illustrates the relationship between the friction force at the attachment surface of the bio-inspired claw–spine and the deformation of the flexible structure. As the normal pre-compression deformation of the flexible structure of the bio-inspired claw–spine increases, the friction force between the claw–spine and the contact surface also increases, and the adjustable range of tangential deformation of the flexible structure becomes larger. When the normal pre-compression displacement of the flexible structure is 2 mm, the adjustable range of tangential deformation is only 1 mm, and the friction force between the claw–spine and the attachment surface is the lowest, with a value of only 0.65 N. When the normal pre-compression displacement of the flexible structure reaches 10 mm, the adjustable range of tangential deformation can increase from 1 mm to 8 mm, and the maximum friction force between the claw–spine and the contact surface reaches 3.88 N. Each experimental trend curve has a corresponding cutoff point. The occurrence of the cutoff point indicates that the claw–spine has detached from the contact surface, meaning that the stable attachment criterion defined by Equation (14) is no longer satisfied.

**Figure 6:**
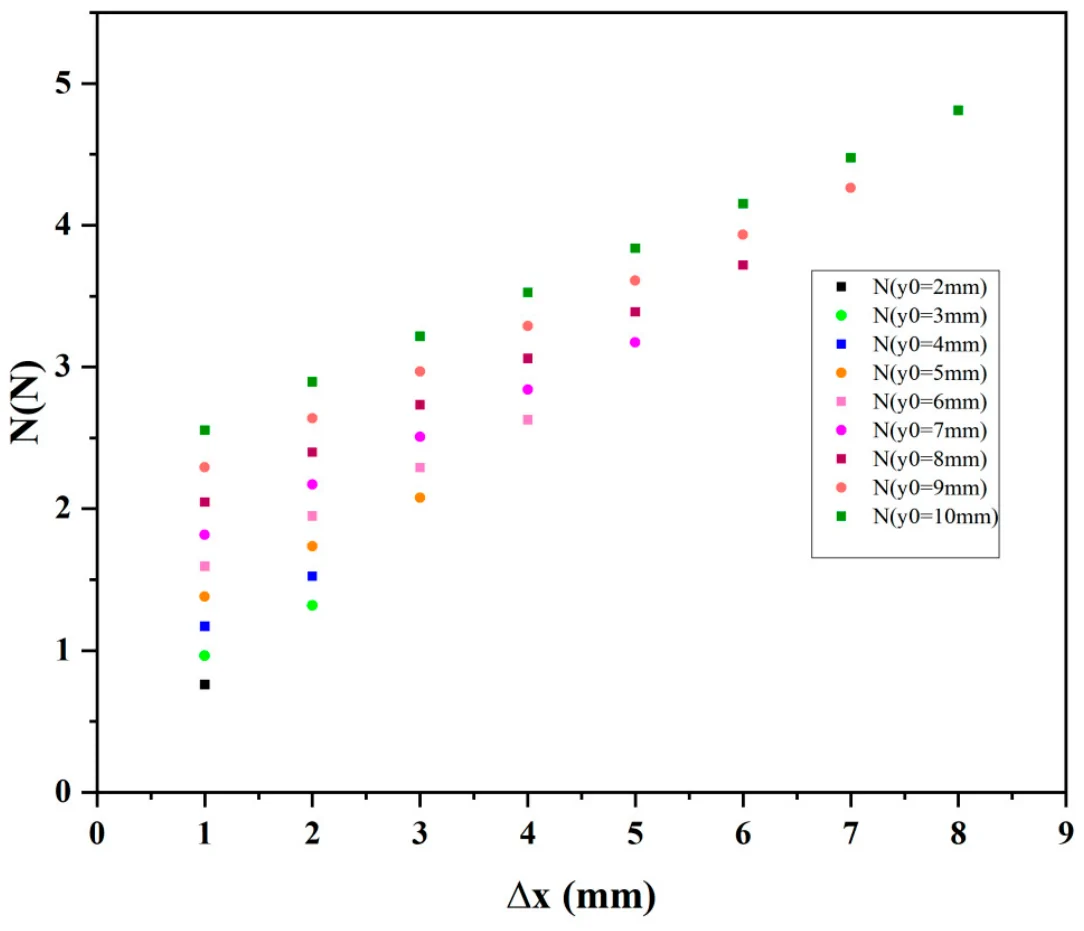
Relationship between normal force at the attachment surface of the bio-inspired claw–spine and deformation of the flexible structure.

Figure 6 shows the relationship between the normal force at the attachment surface of the bio-inspired claw–spine and the deformation of the flexible structure. The normal force acting on the claw–spine is perpendicular to the contact surface and directed downward, preventing the claw–spine from detaching from the contact surface. As the normal pre-compression deformation of the flexible structure increases, the normal force generated between the claw–spine and the contact surface also increases, and the adjustable range of tangential deformation of the flexible structure becomes larger. This indicates that a larger normal pre-compression deformation makes detachment of the claw–spine from the contact surface more difficult. Each experimental trend curve has a corresponding cutoff point. The appearance of the cutoff point indicates that the tangential deformation of the flexible structure can no longer be adjusted, and the claw–spine has detached from the contact surface. At this stage, no contact force is generated between the claw–spine and the surface, and the stable attachment criterion defined by Equation (14) is no longer satisfied.

**Figure 7:**
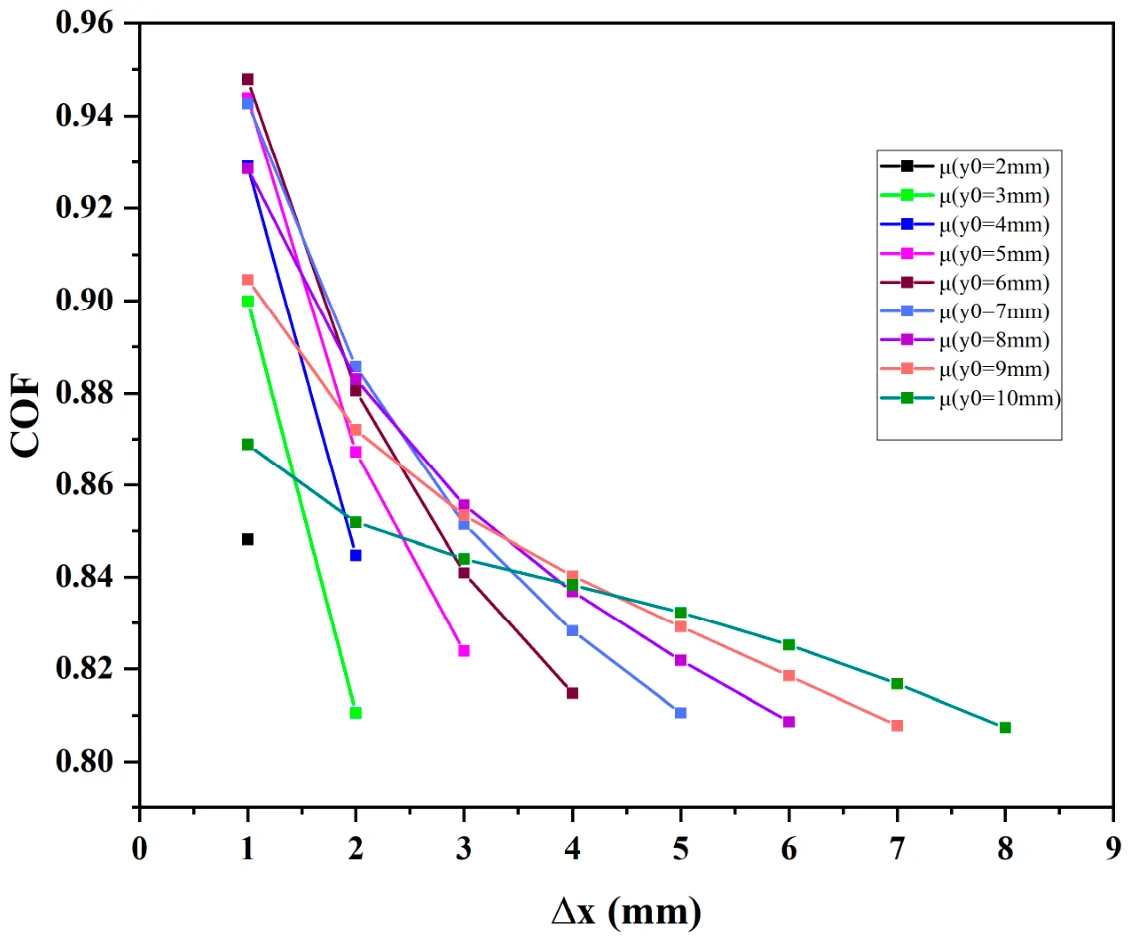
Relationship between the friction coefficient of the bio-inspired claw–spine on a rough surface and deformation of the flexible structure.

Figure 7 shows the relationship between the friction coefficient of the bio-inspired claw–spine on the rough surface and the deformation of the flexible structure. When the normal pre-compression deformation of the claw–spine remains constant, the friction force between the claw–spine and the contact surface decreases with increasing tangential deformation of the flexible structure. Sliding occurs between the claw–spine and the rough contact surface, and the claw–spine eventually detaches from the contact surface when the cutoff point appears. When the tangential deformation of the flexible structure remains constant, the friction coefficient initially increases and then decreases with increasing normal pre-compression deformation. When the pre-compression displacement reaches 6 mm, the friction coefficient reaches a maximum value of 0.95. With further increase in the normal pre-compression displacement, the friction coefficient decreases. This indicates that there is a critical normal pre-compression value for the flexible structure of the claw–spine in the design of future bio-inspired claw–spine wall-climbing robots. At this critical value, the friction force and friction coefficient between the claw–spine and the contact surface can be maximized, thereby improving the stability and reliability of claw–spine attachment.

**Figure 8:**
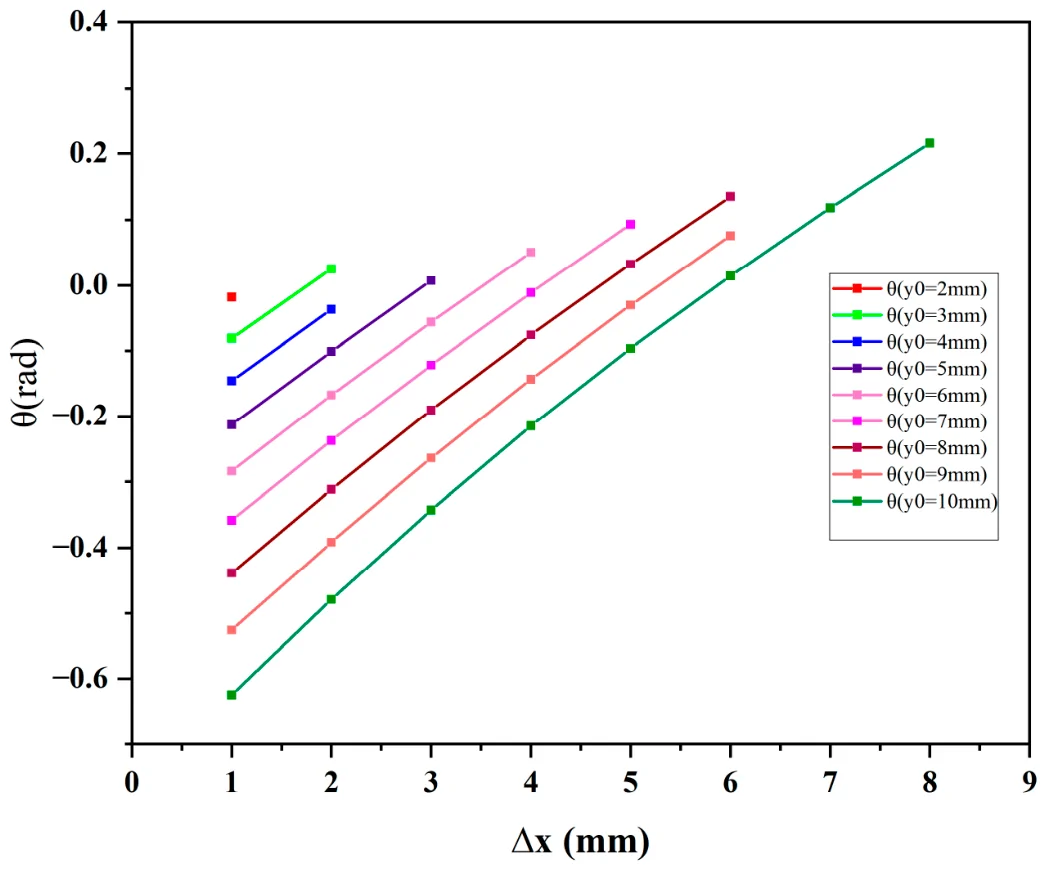
Relationship between the contact angle of the bio-inspired claw–spine at the attachment surface and deformation of the flexible structure.

**Figure 9:**
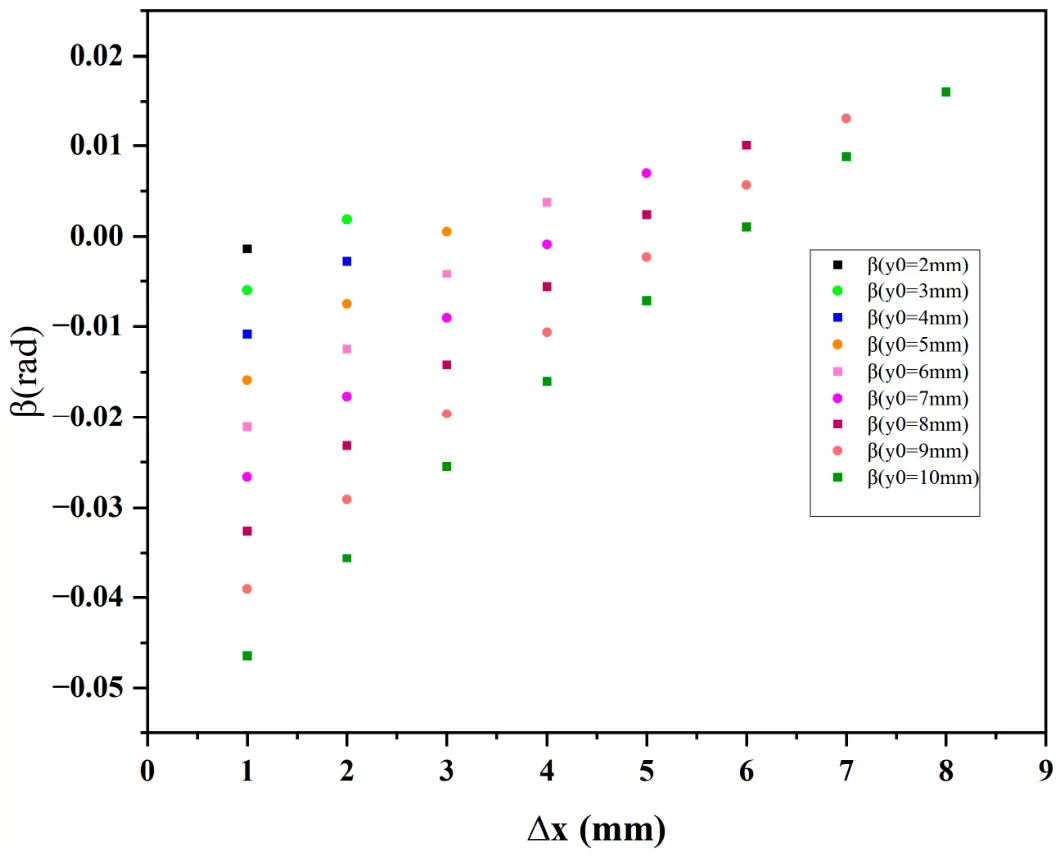
Relationship between the attachment angle of the bio-inspired claw–spine at the attachment surface and deformation of the flexible structure.

Figure 8 shows the relationship between the contact angle of the bio-inspired claw–spine at the attachment surface and the deformation of the flexible structure, while Figure 9 presents the relationship between the attachment angle of the bio-inspired claw–spine and the deformation of the flexible structure. When the normal pre-compression displacement of the flexible structure remains constant, both angles increase with increasing tangential deformation of the bio-inspired flexible claw–spine. Moreover, when the normal pre-compression displacement remains unchanged, the contact angle and attachment angle of the bio-inspired claw–spine on the rough contact surface both increase as the tangential deformation of the flexible structure increases. A larger normal pre-compression displacement results in a wider adjustable range of tangential deformation. Each experimental trend curve has a corresponding cutoff point. The appearance of the cutoff point indicates that the stable attachment criterion defined by Equation (14) is no longer satisfied. At this stage, the tangential deformation of the flexible structure can no longer be adjusted, and the claw–spine has detached from the contact surface, resulting in the loss of attachment and the disappearance of contact force. Because the variation trends of the attachment angle and contact angle of the claw–spine on the contact surface are consistent with the deformation of the flexible structure, only one of these two parameters needs to be considered in the subsequent design of the attachment foot for bio-inspired claw–spine wall-climbing robots. This simplification reduces the design complexity and improves design efficiency.

**Figure 10:**
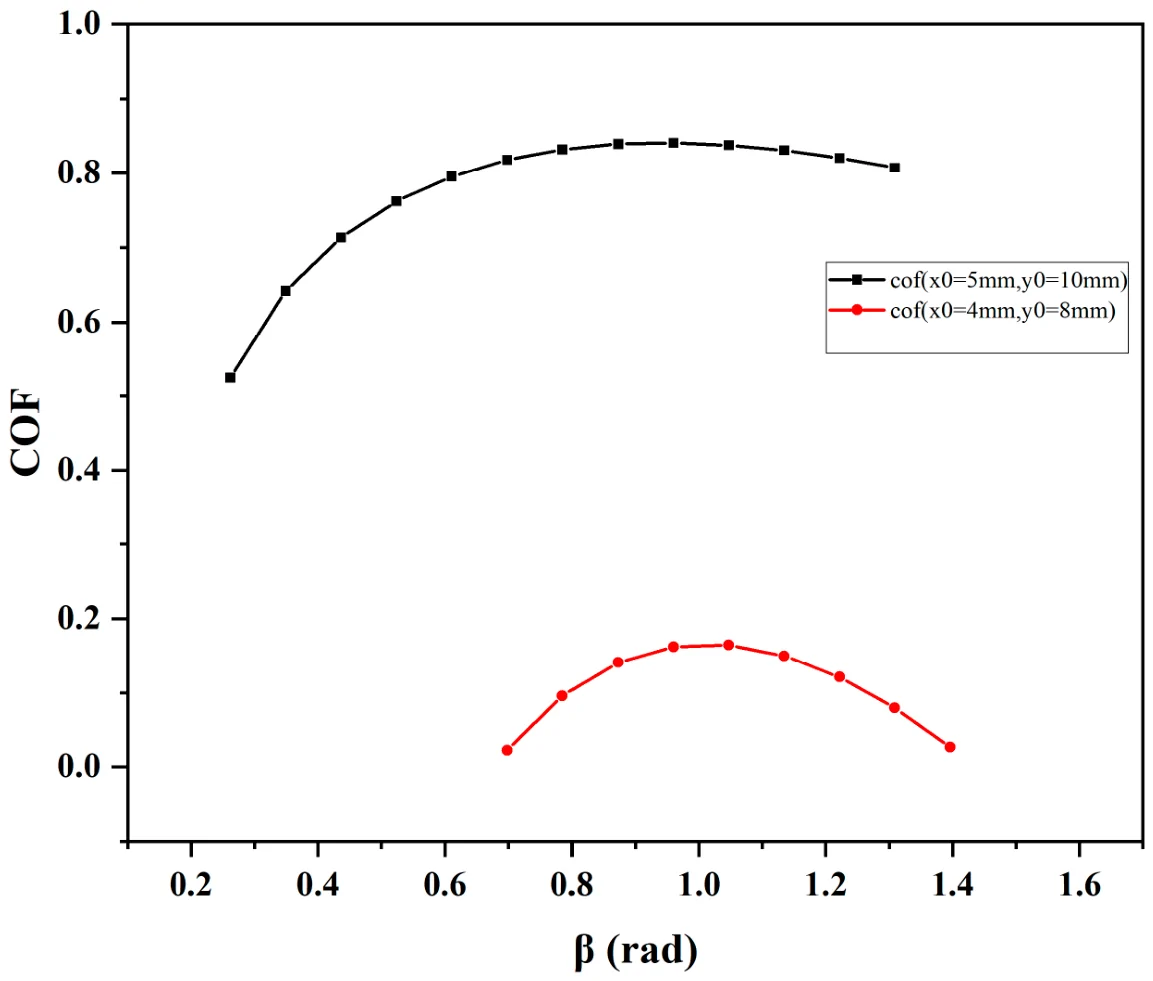
Effect of the attachment angle at the connection point of the flexible claw–spine structure on the friction coefficient at the attachment surface.

The above experimental results indicate that, during the design of bio-inspired claw–spine wall-climbing robots, only one of the attachment angle and contact angle needs to be considered. Therefore, the influence of the claw–spine attachment state on the contact surface was further investigated. Figure 10 shows the effect of the attachment angle at the connection point of the flexible structure of the claw–spine on the friction coefficient at the attachment surface.

The data presented in Figure 4, Figure 5, Figure 6, Figure 7, Figure 8, Figure 9 and Figure 10 are not statistical results obtained from repeated experiments. Instead, they are deterministic parametric calculation results obtained under prescribed geometric parameters, contact parameters, and loading conditions using the contact-mechanics model of a single bioinspired claw–spine structure interacting with an asperity on a rough surface. The calculation method was based on Ref. [36]. Each data point corresponds to a unique numerical solution under a prescribed set of input conditions; therefore, experimental sample sizes, standard deviations, and confidence intervals are not applicable.

During the calculations, the normal precompression displacement was increased from 1 to 10 mm in increments of 1 mm. Under each normal precompression condition, the tangential deformation was also increased from 1 to 10 mm in increments of 1 mm. Only calculation results satisfying the stable-attachment criterion are shown in the figures. The connecting lines between adjacent calculation points indicate the variation trends, whereas curve truncation indicates that the claw–spine structure no longer satisfies the stable-attachment criterion.

In the simulation experiments, the attachment angle varied from 15° to 90° with an increment of 5° for each step, corresponding to an angular range from 0.26 rad to 1.57 rad. Four groups of normal pre-compression displacements and tangential deformations were selected for analysis. Specifically, the cases where the normal pre-compression displacement was twice the tangential deformation and where the tangential deformation was twice the normal pre-compression displacement were considered, including (x_0_ = 5 mm, y_0_ = 10 mm; x_0_ = 4 mm, y_0_ = 8 mm; x_0_ = 10 mm, y_0_ = 5 m; x_0_ = 8 mm, y_0_ = 4 mm). When the tangential deformation of the flexible structure was twice the normal pre-compression displacement, all calculated results failed to satisfy the stable attachment criterion of the claw–spine. Therefore, these results are not presented in Figure 10. The increases in tangential displacement and tangential force are essential for enhancing the normal attachment capacity. Therefore, designing an attachment mechanism that enables controllable regulation of the tangential force is crucial for achieving reliable attachment. When insects attach to a contact surface, their pretarsal claws and spines can form an opposed attachment configuration. For example, the tarsi and distal ends of the tibiae of *Serica orientalis* possess spine-like structures that cooperate with the pretarsal claws to form an opposed attachment configuration, thereby generating redundant constraining forces, increasing the tangential load-bearing capacity, and enhancing the attachment force between the claw–spine foot and the contact surface [37,38,39]. Accordingly, an opposed claw–spine attachment structure was designed in this study based on the biological opposed-attachment principle.

In summary, as the critical tangential displacement increases, the critical normal safe displacement region also expands. When the tangential displacement reaches its maximum value, the critical normal safe pre-compression displacement region reaches its maximum range. However, with further increase in the tangential displacement of the frame, the normal safe displacement region gradually decreases. Specifically, when the tangential displacement variation in the claw–spine remains within the normal safe displacement region, the claw–spine maintains a stable attachment state. When the displacement exceeds this safe region, the claw–spine detaches from the contact surface. Therefore, the increase in tangential displacement and tangential attachment friction force plays a crucial role in enhancing the normal attachment capability of the claw–spine. For different initial contact angles, a larger contact angle results in a larger critical normal safe displacement region and a wider range of allowable tangential displacement variation. Consequently, the stable adjustment range of the claw–spine attachment foot is expanded, leading to improved attachment performance on the contact surface.

## 3. Design of the Bio-Inspired Claw–Spine Wall-Climbing Mechanism and Measurement Platform

### 3.1. Design of the Claw–Spine Attachment Foot

The tarsal chain segments of insects enhance their flexible deformation capability, enabling the claw–spine attachment foot to achieve both attachment and detachment functions. The variations in joint angles among different segments are generally consistent. Based on the above analysis, the design of the flexible claw–spine attachment foot should satisfy the following requirements:

(1) The arrangement of the opposing claw–spines at the end of the claw–spine attachment foot should facilitate the control of individual claw–spines and avoid excessive force concentration on a single claw–spine, which may cause structural damage.
(2) The connection between the claw–spines and the foot substrate should enable sufficient attachment tolerance during the attachment process, allowing the claw–spines to passively adjust their positions.
(3) Each group of flexible connecting structures of the claw–spines should have sufficient independent movement space to prevent interference between adjacent claw–spines.

**Figure 11:**
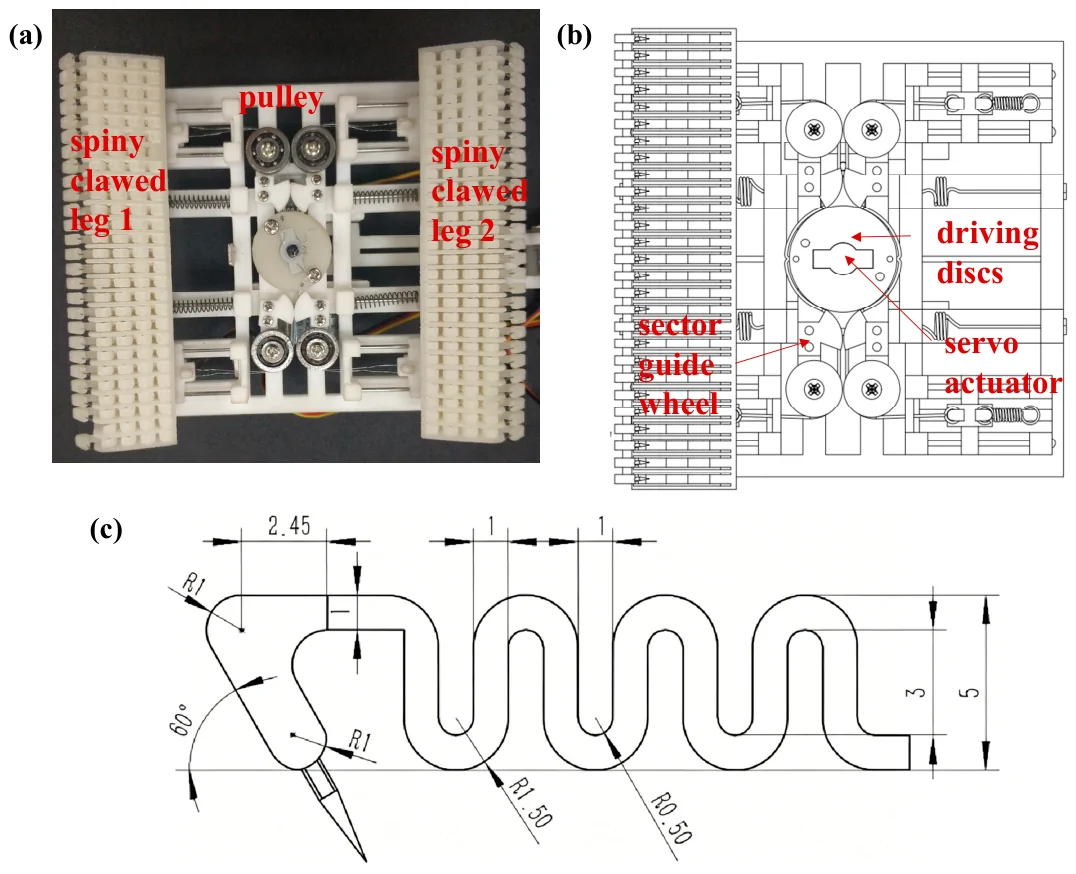
Design of the bio-inspired flexible claw–spine attachment foot. (**a**) Photograph of the bio-inspired flexible claw–spine attachment foot. (**b**) Three-dimensional model of the bio-inspired flexible claw–spine attachment foot. (**c**) Structural design of a single bio-inspired flexible claw–spine.

Based on the friction mechanism of the single claw–spine attachment interface described above, a bio-inspired flexible claw–spine attachment foot was designed, as shown in Figure 11. Figure 11a presents the photograph of the claw–spine opposing attachment foot, Figure 11b shows the three-dimensional model of the claw–spine attachment foot, and Figure 11c illustrates the structural design of a single claw–spine. The principal geometric parameters of this structure were selected with reference to the studies by Liu et al. on spine-based wall-climbing robots, leg–wheel wall-climbing robots, and flexible claw–spine foot structures, while also considering the installation space of the single rigid–flexible claw–spine unit and the arrangement requirements of the subsequent bilateral opposing multi-claw–spine attachment mechanism developed in this study [32,33,40,41]. The main design parameters of the single claw–spine include the overall length *m*, overall height *n*, overall thickness *p*, bending angle of the front section *ζ*, length of the rigid front section *a*, length of each segment in the rear section *b*, inner radius *r* of the S-shaped claw–spine, and outer radius *R* of the S-shaped claw–spine. As shown in Figure 2, the design parameters were set as follows: *m* = 20.15 mm, *n* = 5 mm, *p* = 1.6 mm, *a* = 3.45 mm, *b* = 1 mm, *r* = 0.5 mm, and *R* = 1.5 mm. The diameter of the acupuncture needle used for the claw–spine tip was 0.6 mm. Previous studies have shown that claw–spines can more easily penetrate soft wall surfaces and achieve stable attachment. According to the tribological behavior analysis of the claw–spine attachment interface presented in the previous section, a smaller tip radius of the claw–spine results in stronger attachment capability. The attachment angle of the claw–spine has a significant influence on attachment performance. When this angle ranges from 45° to 60°, the claw–spine exhibits optimal attachment performance and generates a larger normal attachment force. Therefore, the bending angle of the front section of the foot plate was designed as 60°. When the flexible claw–spine structure moves parallel to the wall surface, its orientation angle is 60°, enabling improved attachment performance.

Insects achieve attachment to contact surfaces through the opposing interaction between their terminal claws and spines. For example, *Serica orientalis* possesses spine structures at the ends of its tarsal segments and tibiae, which form an opposing grasping configuration with the tarsal segments. This configuration generates redundant forces to increase the tangential load capacity, thereby enhancing the attachment force between the claw–spine foot and the contact surface. Based on this opposing grasping mechanism, an opposing claw–spine attachment structure was designed. The opposing claw–spine attachment foot mainly consists of two opposing single-foot plates and a driving wheel connected to a servo motor. Each foot segment consists of two single-foot plates, one driving wheel, one wheel cover, four sector-shaped rotating wheels, and four V-groove pulleys. The driving wheel is connected to the servo motor. One end of a flexible steel wire is connected to the driving wheel, while the other end is routed through the rotating wheels and V-groove pulleys and connected to a slider. The slider is connected to the single-foot plate through a spring inside the guide rail of the base. The servo motor drives the spring deformation to achieve the attachment and detachment motions of the claw–spine foot.

The claw–spine attachment foot consists of multiple claw–spine foot plates arranged in an array. A single flexible claw–spine foot can be considered as consisting of three parts: a flexible connecting structure, a rigid connecting structure, and a rigid claw–spine. Among them, the flexible connecting structure in the middle of each claw–spine foot plate adopts a series-connected “S”-shaped configuration. When mechanical interlocking is formed between the claw–spine and the contact surface, the serially connected “S”-shaped structures provide a certain amount of compliant sliding space for the rigid claw–spine, allowing it to adapt to contact surfaces with different surface morphologies.

Insects can control their corresponding claw–spines through the contraction and relaxation of muscles inside the tibial segments of their feet, thereby achieving attachment and detachment. However, in the design of wall-climbing robots, it is difficult to achieve one-to-one control between the driving servo motor and individual claw–spines. Therefore, the opposing claw–spine attachment foot was designed as an array structure consisting of multiple claw–spine foot plates. Each claw–spine foot plate consists of 27 spine plates, and a claw–spine is installed at the end of each spine plate. The spine plates are arranged in a linear array. Chamfers are designed at each bending section of the “S”-shaped structure to facilitate fabrication while improving flexibility and attachment performance. The mounting holes of the rigid claw–spine structures at the ends of the spine plates adopt clearance fits, and the gaps are filled and fixed with polyurethane. By driving the array of claw–spine foot plates, the attachment state of the claw–spines within the array can be indirectly sensed and detected, thereby achieving attachment force regulation.

**Figure 12:**
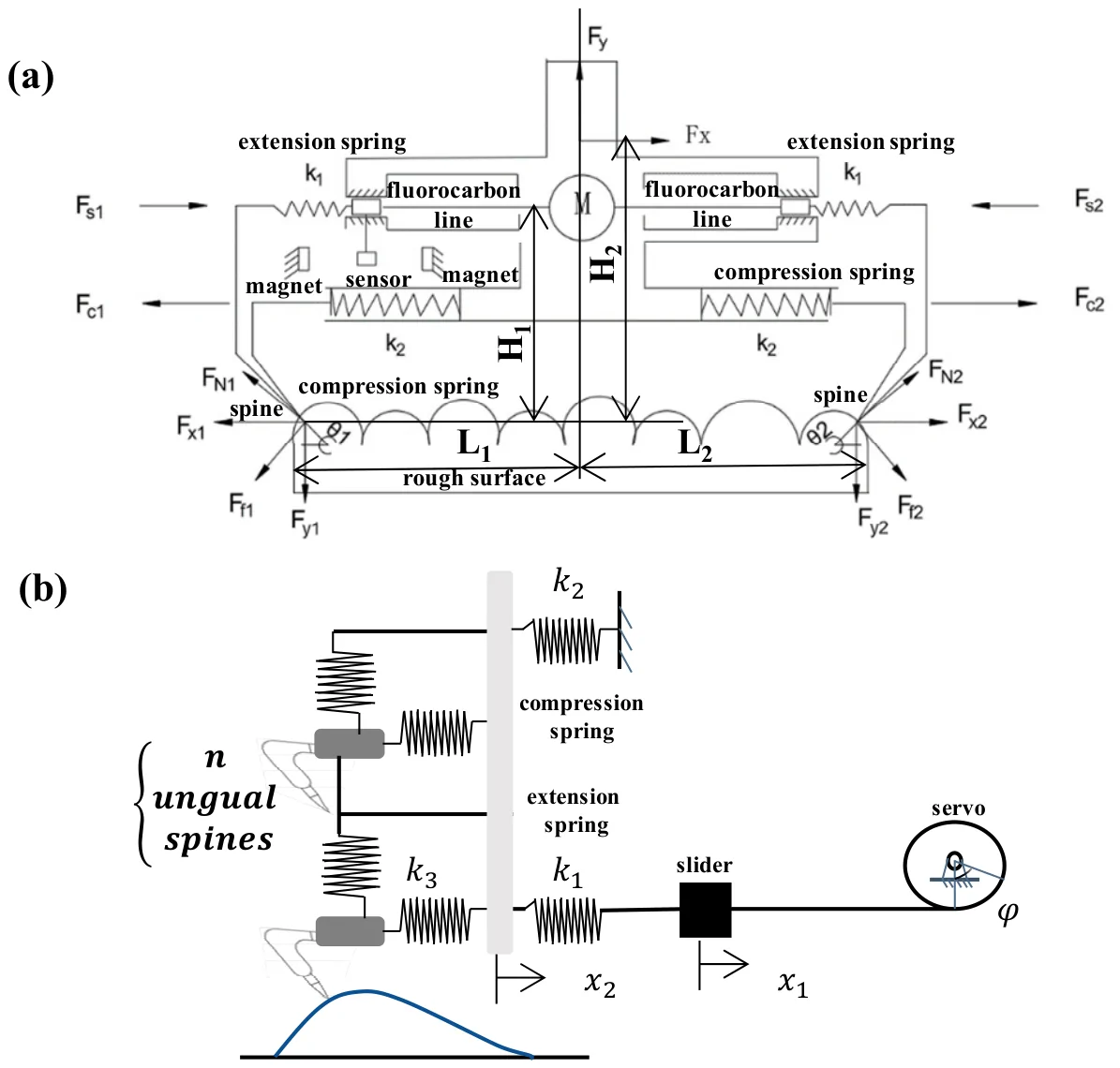
Principle diagram of attachment force control for the bio-inspired flexible claw–spine. (**a**) Force analysis of the opposing claw–spine attachment foot. (**b**) Principle of attachment force control for the bio-inspired flexible claw–spine.

Based on the above design of the opposing claw–spine attachment foot, the attachment mechanism was further investigated to establish the principle of attachment force sensing. The schematic diagram of the driving structure of the claw–spine attachment system is shown in Figure 12: two permanent magnets are installed at the end of each guide rail, and Hall sensors are mounted on the sliders. During slider movement, the Hall sensors detect the displacement caused by spring compression or extension, thereby indirectly determining the attachment force of the claw–spines. Figure 12a illustrates the force analysis of the opposing claw–spine attachment foot. The two opposing claw–spine feet are connected to tension springs and compression springs through flexible steel wires, respectively, and are driven by a circular disk connected to the servo motor. When the servo motor rotates the driving wheel by a certain angle, the two opposing claw–spine feet are subjected to the tensile forces $F_{s1}$ and $F_{s2}$ generated by the tension springs, causing the two tension springs to move toward each other. Meanwhile, the opposing claw–spine feet are also subjected to the supporting forces $F_{c1}$ and $F_{c2}$ from the compression springs, whose directions are opposite to those of the tension forces. In addition, the claw–spines experience friction and normal forces from the contact surfaces. The normal forces perpendicular to the contact surfaces are $F_{N1}$ and $F_{N2}$, while the friction forces parallel to the contact surfaces are $F_{f1}$ and $F_{f2}$. The supporting and friction forces acting on both sides of the opposing claw–spine feet are decomposed into horizontal components $F_{x1}$ and $F_{x2}$, and vertical components $F_{y1}$ and $F_{y2}$. T The surface profiles of the contact asperities on both sides are simplified as spherical curves, and the contact angles between the claw–spines and the surface asperities are defined as $\theta_{1}$ and $\theta_{2}$, respectively.

Taking the left-side claw–spine plate of the opposing attachment foot shown in Figure 12a as an example, the force components can be expressed as:

(18) $$F_{x1}\;=\;F_{N1}\cos\theta_{1}\;+\;F_{f1}\sin\theta_{1}$$

(19) $$F_{x2}=F_{f1}\cos\theta_{1}-F_{N1}\sin\theta_{1}$$

where the force satisfies:

(20) $$F_{f1}=\;{\mu F}_{N1}$$

Combining the above equations gives:

(21) $$F_{y1}=\frac{\mu -\tan\theta_{1}}{1\;+\;\mu \tan\theta_{1}}F_{x1}$$

(22) $$F_{y2}=\frac{\mu -\tan\theta_{2}}{1+\mu \tan\theta_{2}}F_{x2}$$

The resultant force of the tension spring and compression spring is defined as $F_{u}$, which can be expressed as:

(23) $$F_{u}=F_{s1}\;+{\;F}_{c2}-F_{s2}-F_{c1}$$

Taking the left-side claw–spine plate of the opposing claw–spine attachment foot as an example, the force equilibrium in the x-direction can be expressed as:

(24) $$F_{s1\;}=\;F_{c1\;}+\;F_{x1}$$

Similarly, the right-side claw–spine plate satisfies:

(25) $$F_{s2}\;=\;F_{c2}\;+\;F_{x2}$$

Combining Equations (23)–(25), the following relationship can be obtained:

(26) $$F_{u}\;=\;F_{x1}\;+{\;F}_{x2}$$

According to the overall force analysis of the claw–spine attachment foot, the resultant force in the x-direction is:

(27) $$F_{x}\;=F_{x1}-F_{x2}$$

(28) $$F_{y}=F_{y1}-F_{y2}$$

(29) $$F_{x}H_{2}={\;F}_{y2}\left(L_{1}+{\;L}_{2}\right)=F_{y}L_{1}$$

Here, only the forces acting on the foot unit during attachment to a horizontal surface are analyzed

(30) $$F_{x1}={\;F}_{s1}-F_{c1}$$

(31) $$F_{x2}={\;F}_{s2}-F_{c2}$$

By designing a two-stage driving mechanism and measuring $F_{x1}$ and $F_{x2}$, the attachment force state of the opposing claw–spine foot can be indirectly sensed. Based on the above structural design of the opposing claw–spine attachment foot, the forces acting on the attachment foot in the horizontal and vertical directions, $F_{x}$ and $F_{y}$, can be obtained by measuring the elastic forces of the tension and compression springs. According to the above analysis, a larger $F_{y}$ indicates stronger attachment performance of the attachment mechanism. According to Equation (28), increasing the normal attachment force F_y_ of the opposing claw–spine attachment foot requires increasing the two vertical force components $F_{y1}$ and $F_{y2}$. As discussed in the previous section, to increase the vertical force F_y_ of the opposing claw–spine attachment foot, $F_{x1}$ and $F_{x2}$ should be increased when the attachment angle $\theta_{1}$ of the contact surface profile and the friction coefficient μ of the contact surface remain constant. Therefore, the above analysis provides a theoretical basis for the subsequent design of the attachment force detection system. During attachment force experiments, the threshold ranges of $F_{x1}$ and $F_{x2}$ required for stable attachment can be determined.

As shown in Figure 12b, the connection between the claw–spine and the frame is simplified as two elastic elements arranged in the horizontal and vertical directions. The claw–spine attachment foot is connected to the tension spring and compression spring. The claw–spine plates on both sides of the opposing attachment foot are symmetrically arranged, and each single-foot plate is connected to one tension spring and one compression spring. The stiffness coefficients of the tension spring and compression spring are k_1_ and k_2_, respectively. One end of the tension spring is connected to the single-foot plate, while the other end is connected to the slider. The slider is connected to the circular disk through a flexible steel wire and pulley mechanism. The driving wheel is connected to the servo motor. During servo motor rotation, the flexible steel wire drives the linear motion pair composed of the slider and guide rail, thereby causing the tension spring to extend or contract. This movement controls the displacement of the single-foot plate and regulates the contact between the rigid claw–spine at the end of the single-foot plate and the climbing surface, thus achieving attachment and detachment motions. Each tension spring and compression spring is connected to a slider equipped with a Hall sensor. Identical permanent magnets are installed on both sides of each spring. When the spring undergoes deformation, the Hall sensor detects the variation in spring deformation and calculates the corresponding spring force, thereby sensing the state of the tangential force acting on the claw–spine.

The servo motor drives the slider through the flexible steel wire, and the displacement of the slider is $x_{1}$. The slider pulls the frame through the tension spring, resulting in a frame displacement of $x_{2}$. When the claw–spine is in a stable attachment state, the displacement of the horizontal elastic element connected to the claw–spine is $x_{2}$. The stiffness coefficients of the tension spring, compression spring, and horizontal elastic element of the claw–spine are $k_{1}$, $k_{2}$, and $k_{3}$, respectively. The tangential force of a single claw–spine plate is defined as $F_{t}$, and it is assumed that the attachment force of each claw–spine is identical. The number of claw–spines is n. Since the normal force of the claw–spine is proportional to its tangential force, the attachment state of the claw–spines can be evaluated by analyzing the tangential force.

A single claw–spine attachment foot contains two tension springs and two compression springs. It is assumed that both the tension springs and compression springs are initially at their original lengths. When the servo motor rotates by an angle φ, the slider moves a distance $x_{1}$, and the frame moves a distance $x_{2}$.

The force generated by the tension spring is:

(32) $$F_{\mathrm{tension}\;\mathrm{spring}}\;={\;k}_{1}\left(x_{1}-x_{2}\right)$$

The force generated by the compression spring is:

(33) $$F_{\mathrm{compression}\;\mathrm{spring}}\;=\;k_{2}x_{2}$$

According to the force equilibrium of the claw–spine attachment foot:

(34) $$nF_{t}\;=\;2\left(F_{\mathrm{tension}\;\mathrm{spring}}-F_{\mathrm{compression}\;\mathrm{spring}}\right)$$

(35) $$nF_{t}=2\left(k_{1}\left(x_{1}-x_{2}\right)-k_{2}x_{2}\right)$$

For a single claw–spine plate:

(36) $$F_{t}\;=\;k_{3}x_{2}$$

The tangential attachment force of a single claw–spine can be obtained as:

(37) $$F_{t\;}=\;2x_{1}k_{1}/\left({\mathrm{nk}}_{3\;}+\;2\left(k_{1}\;+\;k_{2}\right)\right)$$

The geometric relationship between the servo motor rotation angle and the slider displacement is given as:

(38) $$x_{1}\;=\;\pi \varphi$$

Finally, the relationship between the servo motor rotation angle and the tangential force is obtained as:

(39) $$F_{t}\;=\;2\pi \varphi k_{1}/\left({\mathrm{nk}}_{3}\;+\;2\left(k_{1}\;+{\;k}_{2}\right)\right)$$

Based on the above analysis of the attachment force sensing principle, it is feasible to sense the tangential attachment force by measuring the spring force. By controlling the output rotation angle of the servo motor, the corresponding tangential friction force can be obtained. Meanwhile, the current attachment state can be determined by measuring the spring deformation displacement through the corresponding sensors. According to the detection results, the servo motor rotation angle can be further adjusted to regulate the claw–spine attachment force. Therefore, based on the tribological theory of the claw–spine attachment interface, the proposed method enables attachment force sensing, detection, and control of the claw–spine system.

### 3.2. Design of the Bio-Inspired Claw–Spine Wall-Climbing Robot Mechanism

**Figure 13:**
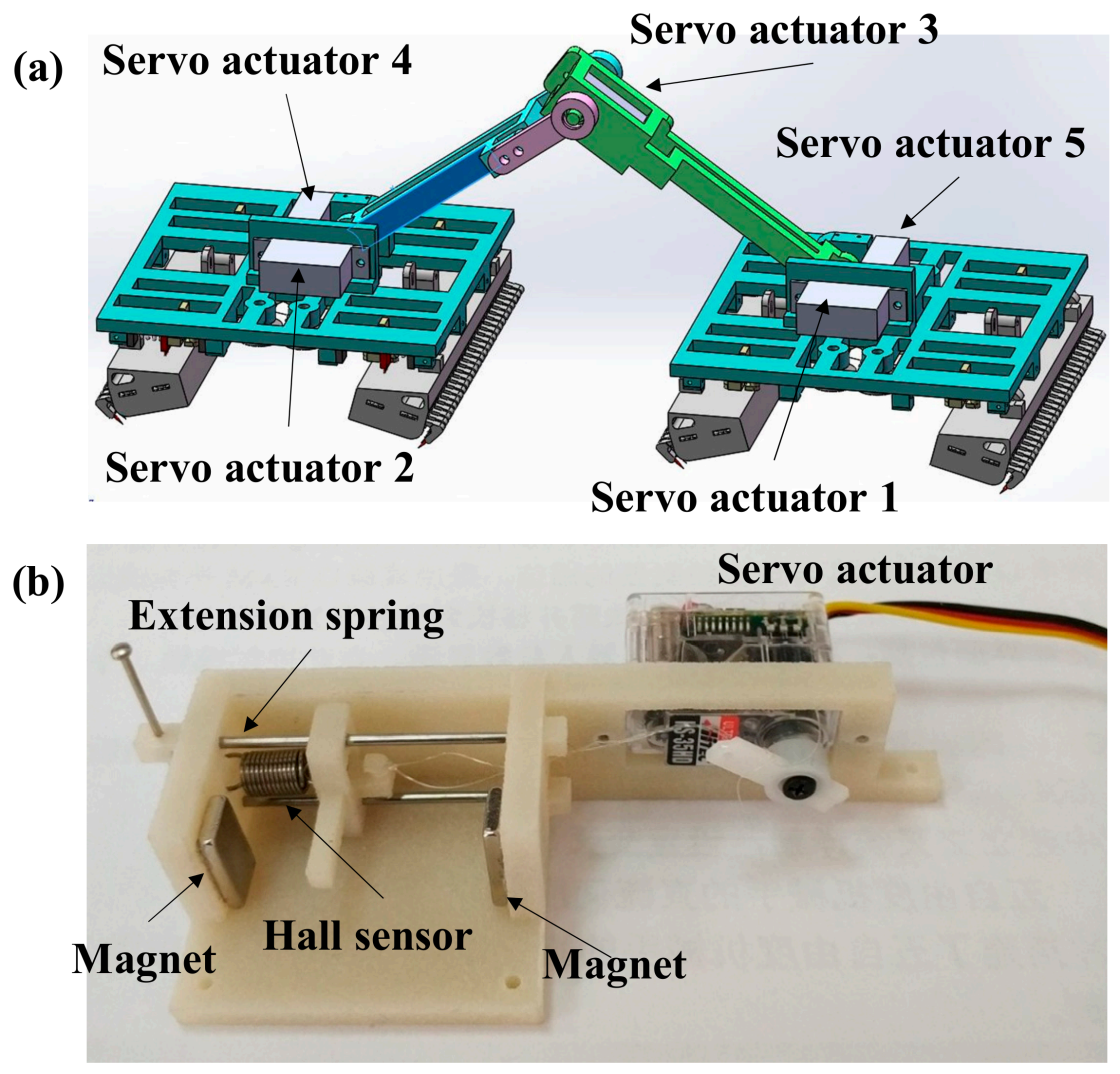
Design of the bio-inspired flexible claw–spine wall-climbing mechanism. (**a**) Three-dimensional model of the bio-inspired flexible claw–spine wall-climbing mechanism. (**b**) Prototype of the claw–spine attachment force measurement validation mechanism.

Figure 13 shows the overall structure of the attachment and locomotion mechanism (hereafter referred to as the locomotion mechanism). Figure 13a presents the three-dimensional model of the claw–spine wall-climbing mechanism. The designed locomotion mechanism has a compact size and complex structure. Conventional machining methods are inefficient and cannot provide sufficient manufacturing accuracy. Therefore, a 3D printing method was adopted for fabrication. The selected 3D printer was the Sinterstation 2500 plus (3D Systems, Inc., Rock Hill, SC, USA), which employs selective laser sintering (SLS) technology based on powder-bed fusion principles, with a manufacturing error range of ±0.04 mm. Nylon was used to fabricate the single-foot plates, base, sliders, driving wheels, and rollers, while photosensitive resin was used to fabricate the servo arms, femoral segments, tibial segments, and driving wheel covers. Nylon was selected as the fabrication material for the flexible connecting structures of the claw–spines, whereas photosensitive resin was used for the rigid connecting structures and connecting rods of the claw–spines.

The selected material properties are listed in Table 1 and Table 2. A commercially available acupuncture needle (Huanqiu brand, Suzhou Acupuncture Products Co., Ltd., Suzhou, China) was selected as the rigid claw–spine element of the flexible claw–spine structure. The locomotion mechanism consists of two connecting rods linked by servo motor 3. This servo motor adjusts the angle between the two connecting rods, thereby controlling the spatial position of the locomotion mechanism. The two connecting rods are connected to the claw–spine attachment feet through two additional servo motors, respectively. The rotation angles of these two servo motors determine the gait transformation and the trajectory of the claw–spine tips. Meanwhile, when the claw–spine feet contact the attachment surface, regulating the rotation angles of these servo motors can increase the attachment force of the claw–spines. Similarly, when the claw–spine feet are in the detachment state, adjusting the corresponding rotation angles facilitates rapid separation of the claw–spine feet from the contact surface.

The total mass of the mechanism is 130 g, with most of the weight concentrated in the two bio-inspired flexible opposing claw–spine attachment feet. The mass of each opposing claw–spine attachment foot is 57 g, while the mass of the connecting rod structure is 16 g. The mechanism is driven by five servo motors. Servo motors 1, 2, and 3 are used for posture adjustment, while servo motors 4 and 5 are used for attachment and detachment actuation. The attachment-drive servo motors control the attachment and detachment motions of the two opposing claw–spine attachment feet, whereas the posture-control servo motors drive the connecting rods to adjust their relative angles, enabling the extension and contraction motions of the connecting rods and posture adjustment during climbing. During forward climbing, servo motor 4 drives the left claw–spine attachment foot to attach to the climbing surface, while servo motor 5 drives the right claw–spine attachment foot to detach from the surface. Servo motors 1, 2, and 3 cooperatively adjust the angles of the connecting rods, causing the mechanism body to contract and lift the right claw–spine attachment foot. Subsequently, servo motors 1, 2, and 3 continue adjusting the connecting rod angles, allowing the mechanism body to extend and lower the right claw–spine attachment foot onto the surface. The right claw–spine attachment foot then attaches to the climbing surface, completing one climbing cycle.

Figure 13b shows the prototype of the bio-inspired claw–spine attachment force testing mechanism. Since the force at the claw–spine tip cannot be directly measured, the proposed tribological mechanism of the claw–spine attachment interface was adopted. An indirect measurement method based on spring force was employed. According to Hooke’s law, the spring deformation was converted into the tangential attachment force generated by the claw–spines, thereby enabling attachment force measurement. Because spring deformation involves micro-displacement measurement, Hall sensors were selected as the sensing elements. Two identical high-strength permanent magnets were arranged with their identical magnetic poles facing each other, leaving a certain gap between them. The two magnets were positioned on the same horizontal plane, and the Hall sensor was placed at the midpoint of the central axis between the two magnets, where the magnetic flux density is theoretically zero. This position was defined as the initial point. When the Hall sensor moves along the central axis by a displacement of ∆z, the sensor outputs a corresponding voltage signal, and the output voltage is proportional to the displacement variation in the Hall sensor. The Hall effect refers to the phenomenon that when a metal or semiconductor thin film carrying current is placed perpendicular to a magnetic field, a transverse potential difference is generated across the two sides of the film, producing an electromotive force perpendicular to both the magnetic field and the current direction.

The Hall output voltage is proportional to the product of the input current and magnetic flux density. Under constant temperature conditions, when a constant current is applied, the Hall output voltage increases with increasing magnetic flux density. When a linear Hall sensor undergoes displacement within a magnetic field with a magnetic flux density of B, the magnetic flux density changes approximately linearly with the increase or decrease in sensor displacement, resulting in a corresponding linear variation in the Hall output voltage. By detecting the voltage variation caused by the displacement of the Hall sensor, the claw–spine attachment force can be estimated. When the servo motor drives the deformation of the spring connected to the claw–spine, the Hall sensor connected to the spring moves synchronously within the magnetic field and generates a corresponding displacement. According to the Hall effect, the spring deformation can be obtained, and the claw–spine attachment force can then be calculated. Therefore, the attachment state of the claw–spine can be determined, enabling the self-sensing capability of the robot’s claw–spine frictional attachment force.

**Figure 14:**
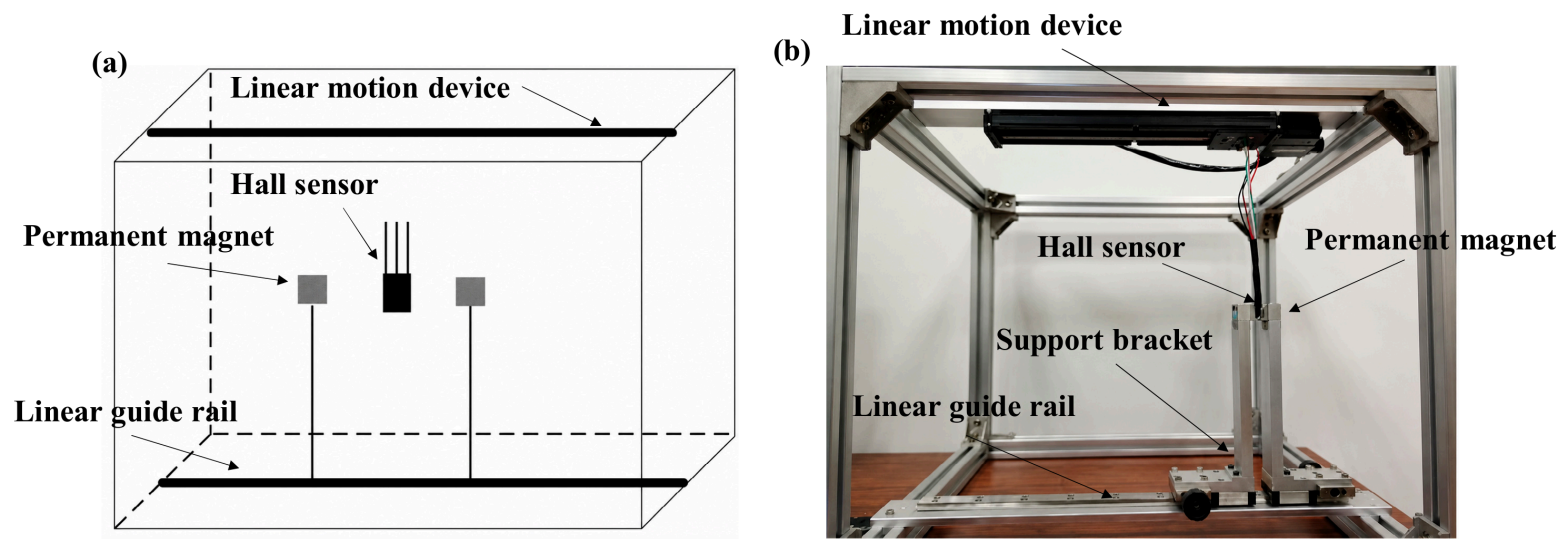
Hall-sensor displacement calibration platform: (**a**) schematic diagram; (**b**) photograph of the experimental setup.

The attachment-force detection system was designed based on a Hall sensor. Because the accuracy of distance measurement by the sensor directly affects the measurement results of the claw–spine attachment force, the relationship between Hall-sensor displacement and output voltage was first calibrated. Since the displacement of the Hall sensor is caused by changes in the servo-motor rotation angle, the relationship between servo-motor angle and displacement was then calibrated based on the displacement–voltage calibration results. Finally, the relationship between servo-motor angle and attachment force was determined. The resulting calibration relationship can be used for attachment-force detection and control. An experimental platform was first established to determine the relationship between the Hall-sensor output voltage and the linear displacement of the Hall sensor. The test platform is shown in Figure 14.

The displacement-measurement accuracy of the Hall sensor directly affects the measured claw–spine attachment force. Therefore, the relationship between the displacement measured by the Hall sensor and the actual travel distance was first calibrated. The calibration platform mainly consisted of a linear translation stage, an aluminum-alloy frame, supports, a guide rail, and a locking mechanism. The guide rail was installed at the bottom of the aluminum-alloy frame, and two permanent magnets were fixed by the supports. The Hall sensor was mounted on the slider of the linear translation stage and could move steadily along the guide rail. During the experiment, prescribed travel distances were applied to the slider, and the corresponding displacements measured by the Hall sensor were recorded. The calibration results are presented in Table 3. Based on the experimental results, the displacement-measurement correction coefficient was set to 0.96 to reduce the error associated with Hall-sensor displacement measurement.

The spring constants of the extension and compression springs were subsequently determined. Forces of 0.005, 0.01, 0.02, 0.03, 0.04, and 0.05 N were applied to the extension and compression springs on the linear translation stage, respectively. After the springs reached a stable deformation state, the corresponding spring deformations were recorded from the linear translation stage. The spring constants of the extension and compression springs were denoted by k_1_ and k_2_, respectively. After weighted averaging of the experimental data, the spring constants were determined to be approximately 0.36 for the extension spring and 0.25 for the compression spring.

To establish the relationship between the Hall-sensor output voltage and spring deformation, an SS495A1 Hall sensor (Honeywell, Fort Mill, SC, USA) was mounted on the slider of the linear translation stage and positioned between two oppositely arranged permanent magnets of the same model and dimensions. The distance between the two permanent magnets was set to 20 mm and fixed using a locking mechanism. As the Hall sensor moved with the slider, its displacement was converted into a voltage signal and transmitted to the host computer through the measurement system.

The power-supply module provided the Hall sensor with a 5 V DC voltage, and the sensor output was connected to the ADC acquisition circuit through a signal-conditioning module. At the beginning of the experiment, the Hall sensor was moved close to the permanent magnet on the far right. At this position, the initial output voltage recorded by both the host computer and the LCD was 0.02 V. The Hall sensor was then moved from right to left in increments of 1 mm over a total travel distance of 20 mm, and the output voltage corresponding to each displacement was recorded. The results are presented in Table 4.

After completing the calibration of the Hall-sensor displacement measurement, the output voltage–displacement relationship, and the spring constants, a further calibration experiment was conducted to establish the relationship between the servo-motor rotation angle and the spring force. In the experiment, the linear Hall sensor was connected to an extension spring with a known spring constant. When the servo motor drove the spring to deform, the Hall sensor moved synchronously with the spring and generated a corresponding voltage signal. The spring deformation was calculated using the calibrated voltage–displacement relationship, and the spring force was subsequently determined from the spring constant. In this manner, the relationship between the servo-motor rotation angle and the spring force was established.

A measurement system based on an STM32 microcontroller was developed. The power supply module of the system provides operating voltage to the analog servo motor HS-35HD and the Hall sensor, respectively. During servo motor actuation, the flexible steel wire drives the spring to stretch. Since the Hall sensor is connected to the spring, it moves synchronously with the deformation of the tension spring. After signal conditioning, the output voltage of the Hall sensor is transmitted to the ADC acquisition module. The displacement variation in the Hall sensor caused by spring extension is obtained through a fitted calibration function, and the displacement of the tension spring is subsequently determined. The experimental data are listed in Table 5. Since the displacement of the tension spring is equal to that of the Hall sensor, the spring deformation can be obtained directly. According to Hooke’s law, the elastic force of the tension spring can then be calculated.

The experimental data were fitted using polynomial regression. The results showed that the quadratic polynomial model provided a good fitting performance. The relationship between the servo rotation angle and the spring force was obtained as follows:

(40) $$y\;=\;m_{1}x^{2}\;+\;m_{2}x\;+\;m_{3}$$

where $m_{1}\;=\;0.00011$, $m_{2}\;=\;0.002791$, $m_{3}\;=\;-0.01296$. The fitting error was 0.007743, the coefficient of determination was 0.9871, the adjusted coefficient of determination was 0.9854, and the standard deviation was 0.02272. Equation (40) describes the relationship between the servo-motor rotation angle and the spring force. By combining the calibrated Hall-sensor output voltage–displacement relationship, the displacement-measurement correction coefficient, and the spring constant, the relationship between the servo-motor rotation angle and the claw–spine attachment force can be further determined and used for attachment-force measurement and adjustment.

### 3.3. Experimental Platform Construction for Attachment Force Measurement of the Bio-Inspired Claw–Spine Wall-Climbing Mechanism

**Figure 15:**
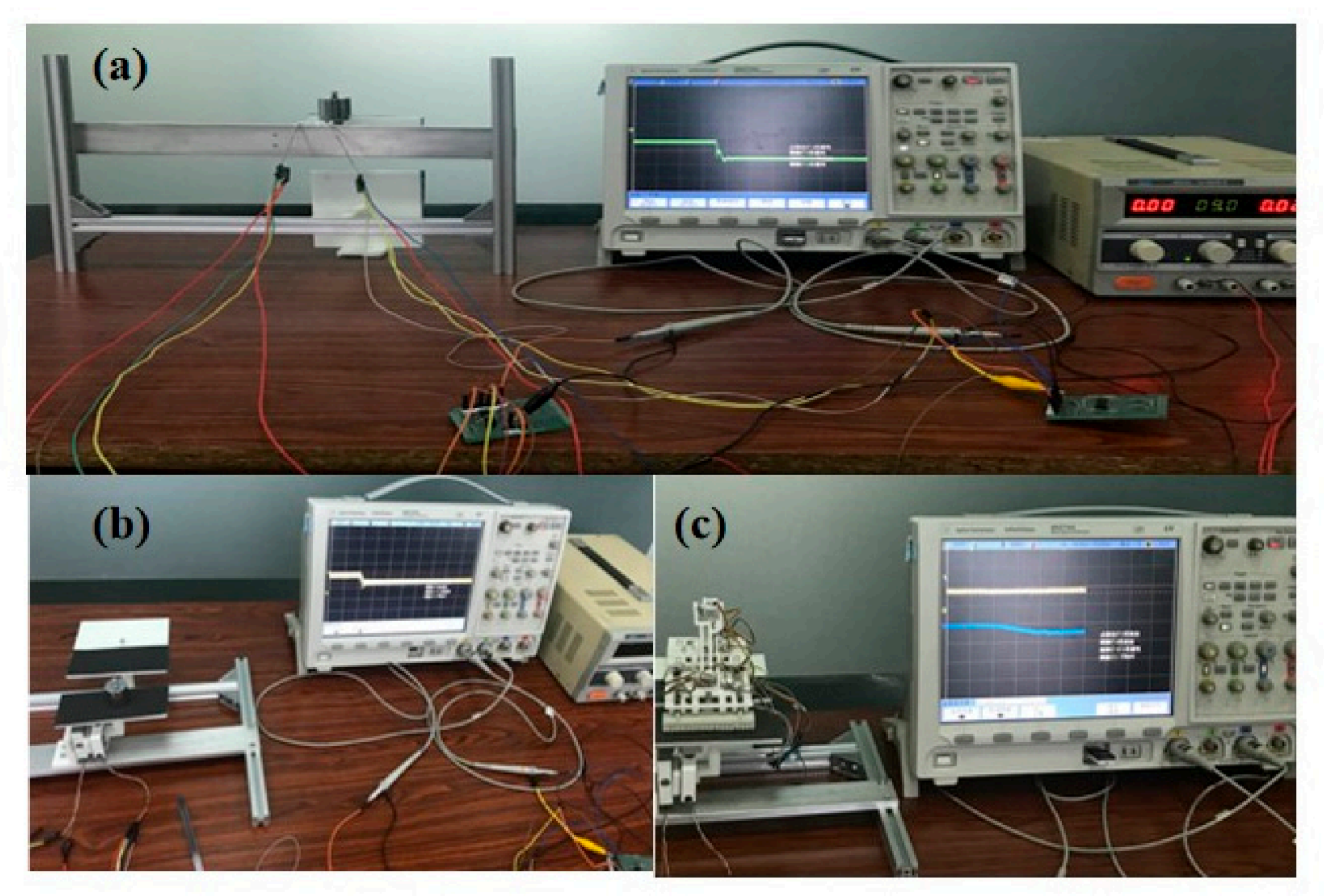
Experimental platform for measuring the attachment force of the bio-inspired flexible claw–spine system. (**a**) Calibration of the measurement platform. (**b**) Sandpaper-based testing platform. (**c**) Example of measured attachment force of the bio-inspired flexible claw–spine system.

To verify the accuracy of the proposed attachment force measurement and control system, the attachment force measured by the Hall sensor-based detection system was compared with that obtained using a standard force sensor. Therefore, an experimental platform equipped with a standard force sensor was constructed for measuring the claw–spine attachment force. As shown in Figure 15, the constructed attachment force measurement platform mainly consists of an aluminum alloy frame. An acrylic plate, which was used as the contact substrate for claw–spine friction attachment experiments, was installed on the metal frame. The force sensor used in this study was an LSM300 tension–compression load cell (FUTEK Advanced Sensor Technology, Inc., Irvine, CA, USA), with a measurement range of 9.8 N, rated output of 2 mV/V, hysteresis of ±0.02% R.O., excitation voltage of 18 V, and operating temperature range of 50–93 °C. A regulated power supply was used to provide stable voltage for the oscilloscope and force sensor. The oscilloscope was used to monitor the variation in claw–spine attachment force under different surface conditions and record the experimental data. Different experimental environments were obtained by placing sandpapers with different roughness levels on the acrylic plate, as shown in Figure 15.

**Figure 16:**
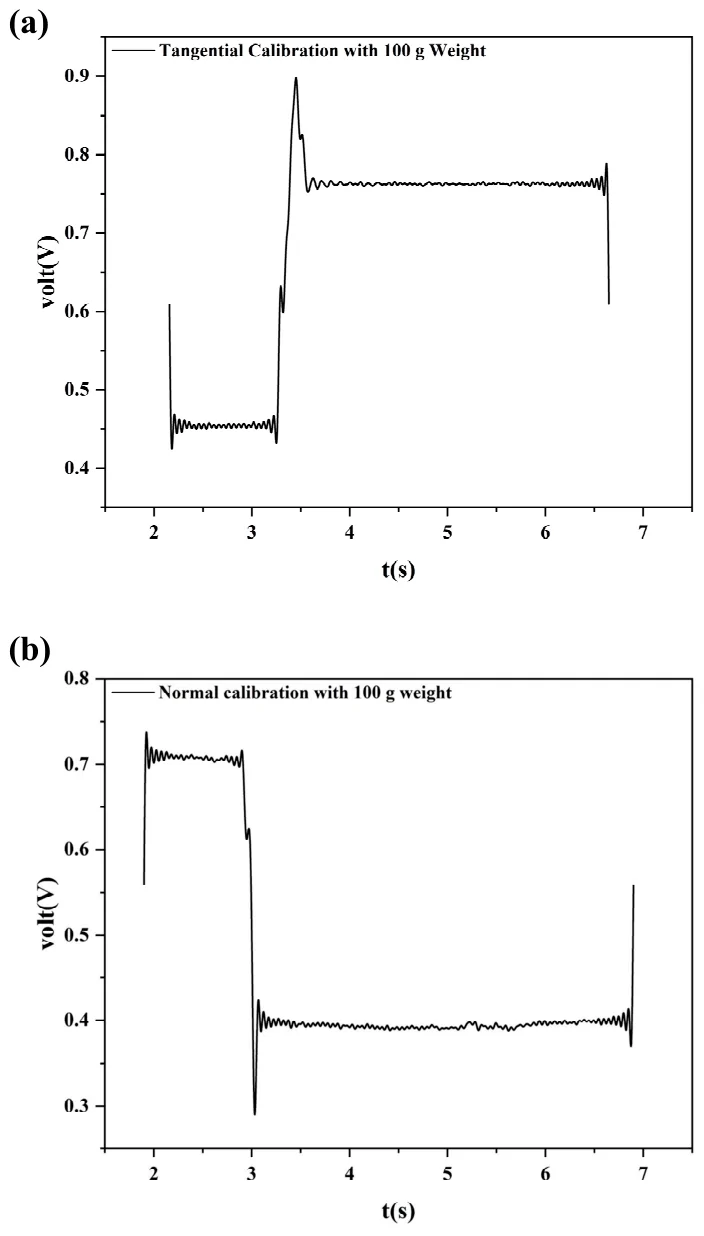
Mechanical calibration of commercial sensors: (**a**) Tangential Calibration; (**b**) Normal Calibration.

The LSM300 force sensor directly outputs voltage signals; therefore, calibration was required to establish the relationship between the output voltage and applied force. In the calibration experiment, the applied force was generated by adding weights. The experimental platform was placed vertically and horizontally to perform normal and tangential calibration, respectively. Standard weights of 100 g were sequentially applied to the tangential sensing direction of the tension–compression load cell, and the corresponding changes in sensor output voltage were recorded. The force calibration was completed based on the measured voltage variations. The calibration results are shown in Figure 16, where Figure 16a presents the tangential calibration results and Figure 16b presents the normal calibration results.

## 4. Results and Analysis

### 4.1. Finite Element Validation of a Single Bio-Inspired Flexible Claw–Spine

To verify the deformation coordination capability and stiffness characteristics of a single bio-inspired flexible claw–spine under attachment loads, a three-dimensional model of a single claw–spine was established in SolidWorks 2025 and imported into the static structural analysis module of ANSYS Workbench 2024R2 for finite element analysis. By applying displacements in the X and Y directions and rotation around the Z-axis at the claw–spine tip, the total deformation, equivalent elastic strain, equivalent stress, reaction force, and reaction moment under different deformation conditions were obtained. The equivalent stiffness matrix was subsequently calculated based on these results.

**Figure 17:**
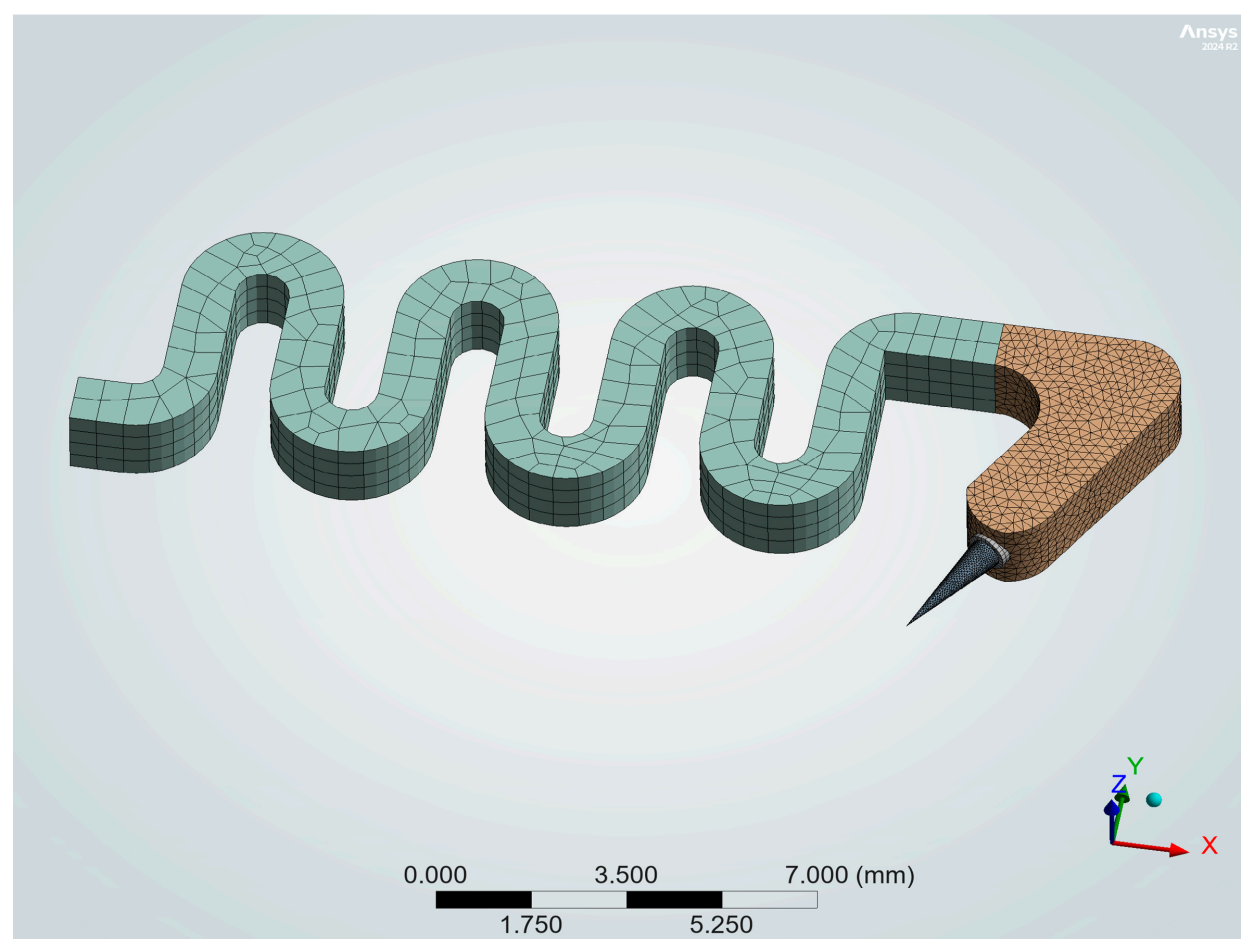
Finite element mesh model of a single bio-inspired flexible claw–spine.

A single bio-inspired flexible claw–spine consists of an S-shaped flexible connector, a nylon rigid connector, a stainless-steel rigid claw–spine, and an annular adhesive layer. The flexible connector is used to accommodate external displacement and coordinate the posture adjustment of the claw–spine, while the rigid connector and rigid claw–spine are mainly responsible for load transmission and attachment support. The adhesive layer is introduced to simulate the bonding fixation between the acupuncture needle and the inner wall of the nylon hole. The flexible connector was made of VYtaFleX 20 polyurethane rubber, and its material parameters were selected with reference to related studies and the official technical data provided by Smooth-On [36,42]. VYtaFleX 20 has a Shore hardness of 20 A, a specific gravity of 1.00 g/cm^3^, a tensile strength of 200 psi, a 100% modulus of 50 psi, and an elongation at break of 1000%. Here, the 100% modulus refers to the tensile stress corresponding to 100% elongation. Because complete hyperelastic constitutive parameters for this material were unavailable, an isotropic linear-elastic material model was adopted in the present numerical analysis. The value of 50 psi was converted to 0.345 MPa and used as the equivalent linear-elastic modulus of the flexible connector, while the Poisson’s ratio was set to 0.49. The material properties and mesh sizes of other components are listed in Table 6. The mesh-generation strategy used in the finite element analysis was established with reference to related studies [32,33]. Different mesh sizes were assigned to the flexible connector, rigid connector, acupuncture needle, and adhesive layer according to the dimensions and structural characteristics of the different model components. Specifically, the mesh sizes were set to 0.5 mm for the flexible connector, 0.2 mm for the nylon rigid connector, 0.05 mm for the acupuncture needle, and 0.1 mm for the adhesive layer. The finite element mesh model of a single bio-inspired flexible claw–spine is shown in Figure 17. The rear mounting surface of the model was defined as a fixed support, and displacements in the X and Y directions, together with a rotation about the Z-axis, were applied at the tip of the claw–spine to simulate the multi-directional deformations induced by contact between the claw–spine tip and a rough surface.

Three deformation levels were defined for each loading mode: displacements of 0.5, 1.0, and 1.5 mm were applied in the X and Y directions, respectively, and rotations of 0.5°, 1.0°, and 1.5° were applied about the Z-axis. Accordingly, a total of nine independent operating conditions were established, as listed in Table 7. All displacement and rotation inputs were applied in the negative directions. The signs of the corresponding reaction forces, reaction moments, and input variables were retained during the stiffness matrix calculation.

**Figure 18:**
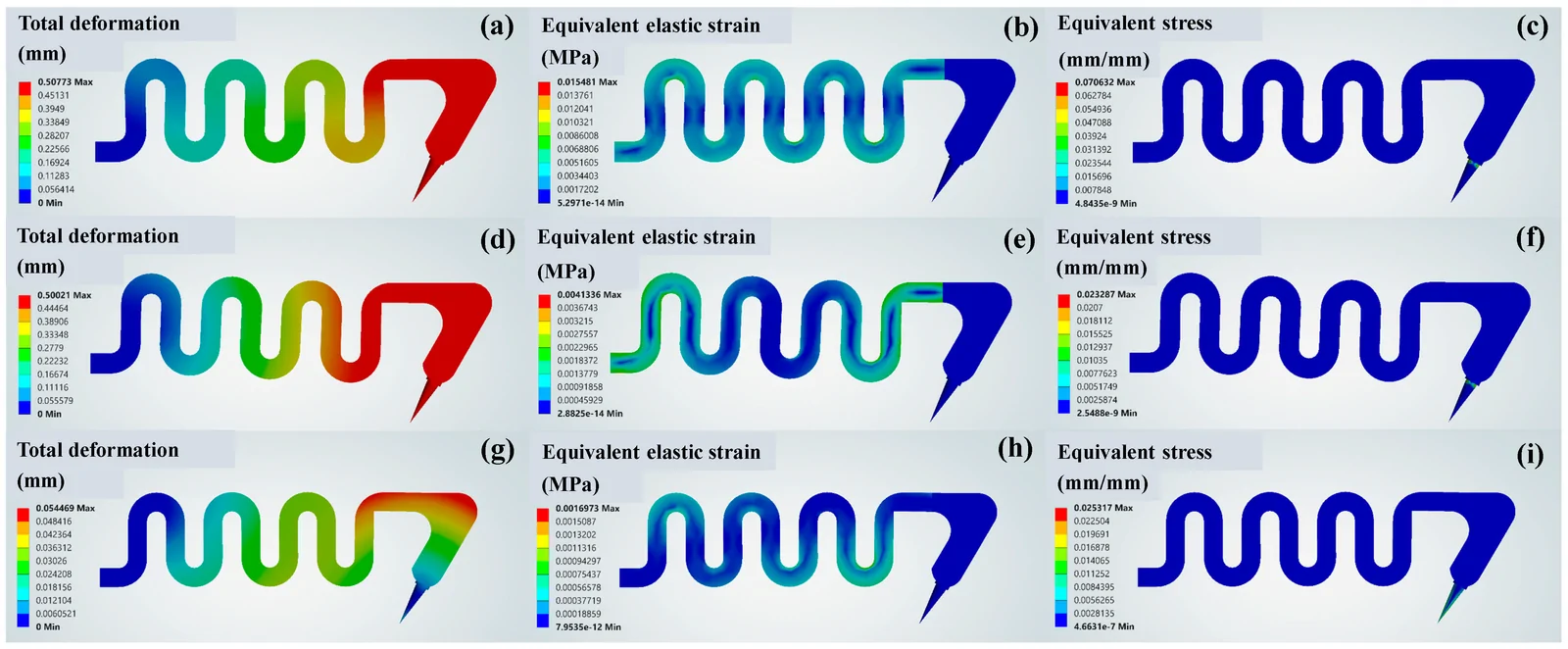
Contour plots of total deformation, equivalent elastic strain, and equivalent stress under conditions of 0.5 mm displacement and 0.5° rotation. (**a**–**c**) Contour plots of total deformation, equivalent elastic strain, and equivalent stress under a 0.5 mm displacement along the x-axis, respectively; (**d**–**f**) contour plots of total deformation, equivalent elastic strain, and equivalent stress under a 0.5 mm displacement along the y-axis, respectively; (**g**–**i**) contour plots of total deformation, equivalent elastic strain, and equivalent stress under a 0.5° rotation.

After solving each operating condition, the maximum total deformation, maximum equivalent elastic strain, and maximum equivalent stress were extracted, and the results are summarized in Table 8. Total deformation represents the displacement magnitude of each node relative to its initial position and primarily reflects the overall deformation behavior of the claw–spine structure. However, local deformation characteristics and stress concentrations cannot be fully evaluated solely from total deformation; therefore, the equivalent elastic strain and equivalent stress contour plots were further analyzed. Figure 18 presents the contour plots of total deformation, equivalent elastic strain, and equivalent stress under the condition of 0.5 mm displacement and 0.5° rotation.

Under X-direction displacement loading conditions, the maximum total deformation increased from 0.50773 mm to 1.5237 mm, the maximum equivalent elastic strain increased from 0.015481 to 0.046116, and the maximum equivalent stress increased from 0.070632 MPa to 0.20112 MPa. All three parameters increased approximately linearly with increasing displacement amplitude. A similar trend was observed under Y-direction displacement loading conditions; however, the peak strain and stress values at the same displacement levels were significantly lower than those under X-direction loading, indicating that the S-shaped flexible connector exhibits pronounced directional mechanical characteristics.

When the rotation angle about the Z-axis increased from 0.5° to 1.5°, the maximum total deformation increased from 0.054469 mm to 0.16341 mm, and the maximum equivalent elastic strain increased from 0.0016973 to 0.0051066, both showing an approximately linear increase. In contrast, the maximum equivalent stress varied from 0.017174 to 0.025896 MPa without a clear monotonic trend, indicating that the stress response under rotational loading is strongly influenced by local stress concentration regions.

The equivalent stiffness matrix was calculated based on the reaction forces and reaction moments obtained under different loading conditions. The extracted results are presented in Table 9. For each deformation level, the X-direction reaction force, Y-direction reaction force, and reaction moment about the Z-axis were obtained from the corresponding X-direction displacement, Y-direction displacement, and Z-axis rotation loading cases, respectively. The stiffness matrix was then calculated according to Equations (15) and (16).

In the equation, F_x_ is the reaction force in the X direction, F_y_ is the reaction force in the Y direction, and M is the reaction torque about the Z-axis; dx is the displacement in the X direction, dy is the displacement in the Y direction, and dα is the angle of rotation about the Z-axis.

Based on the reaction forces and reaction moments listed in the table, the stiffness matrices under conditions a, b, and c were calculated as follows:

$$K_{0.5}\;=\;\left(\begin{array}{lll} {6.094\;\times \;10}^{-4} & {1.787\;\times \;10}^{-6} & -{2.309\;\times \;10}^{-3}\\ {3.424\;\times \;10}^{-5} & {3.202\;\times \;10}^{-6} & {-6.591\;\times \;10}^{-4}\\ {-2.244\;\times \;10}^{-3} & {-6.192\;\times \;10}^{-4} & 1.424{\;\times \;10}^{-2} \end{array}\right)$$

$$K_{1}\;=\;\left(\begin{array}{lll} 6.045{\;\times \;10}^{-4} & {1.555\;\times \;10}^{-5} & {-3.372\;\times \;10}^{-3}\\ {3.212\;\times \;10}^{-5} & {6.221\;\times \;10}^{-5} & -{2.603\;\times \;10}^{-3}\\ {-2.193\;\times \;10}^{-3} & {-5.828\;\times \;10}^{-4} & {1.433\;\times \;10}^{-2} \end{array}\right)$$

$$K_{1.5}\;=\;\left(\begin{array}{lll} 5.997{\;\times \;10}^{-4} & {5.397\;\times \;10}^{-6} & -{2.334\;\times \;10}^{-3}\\ {3.001\;\times \;10}^{-5} & {6.247\;\times \;10}^{-5} & -{6.634\;\times \;10}^{-4}\\ -{2.143\;\times \;10}^{-3} & {-5.488\;\times \;10}^{-4} & {1.441\;\times \;10}^{-2} \end{array}\right)$$

The stiffness matrices under different deformation levels indicate that the primary stiffness components of the single bio-inspired flexible claw–spine remain relatively stable. Specifically, k_XX_ remains approximately 6.0 × 10^−4^ N/mm under the three deformation levels, while k_αα_ remains approximately 1.4 × 10^−2^ N·mm/rad. This indicates that the flexible claw–spine structure maintains stable translational stiffness in the X direction and rotational stiffness within the investigated deformation range. The positive and negative signs of the matrix elements indicate the directional relationship between the reaction forces/moments and the corresponding displacements/rotations.

### 4.2. Hall Sensor—Test Results for Attachment Force Measurement of a Bio-Inspired Flexible Claw-Spine in a Detection System

The Hall-sensor-based attachment-force measurement system connects the prototype signal output to an STM32 microcontroller through a signal-conditioning circuit and subsequently to a computer, with the measurement data displayed in real time. The tangential attachment force of the claw–spine mechanism was measured under unloaded conditions and on 60-grit, 80-grit, and 120-grit sandpaper, as well as on horizontal and vertical brick surfaces.

**Figure 19:**
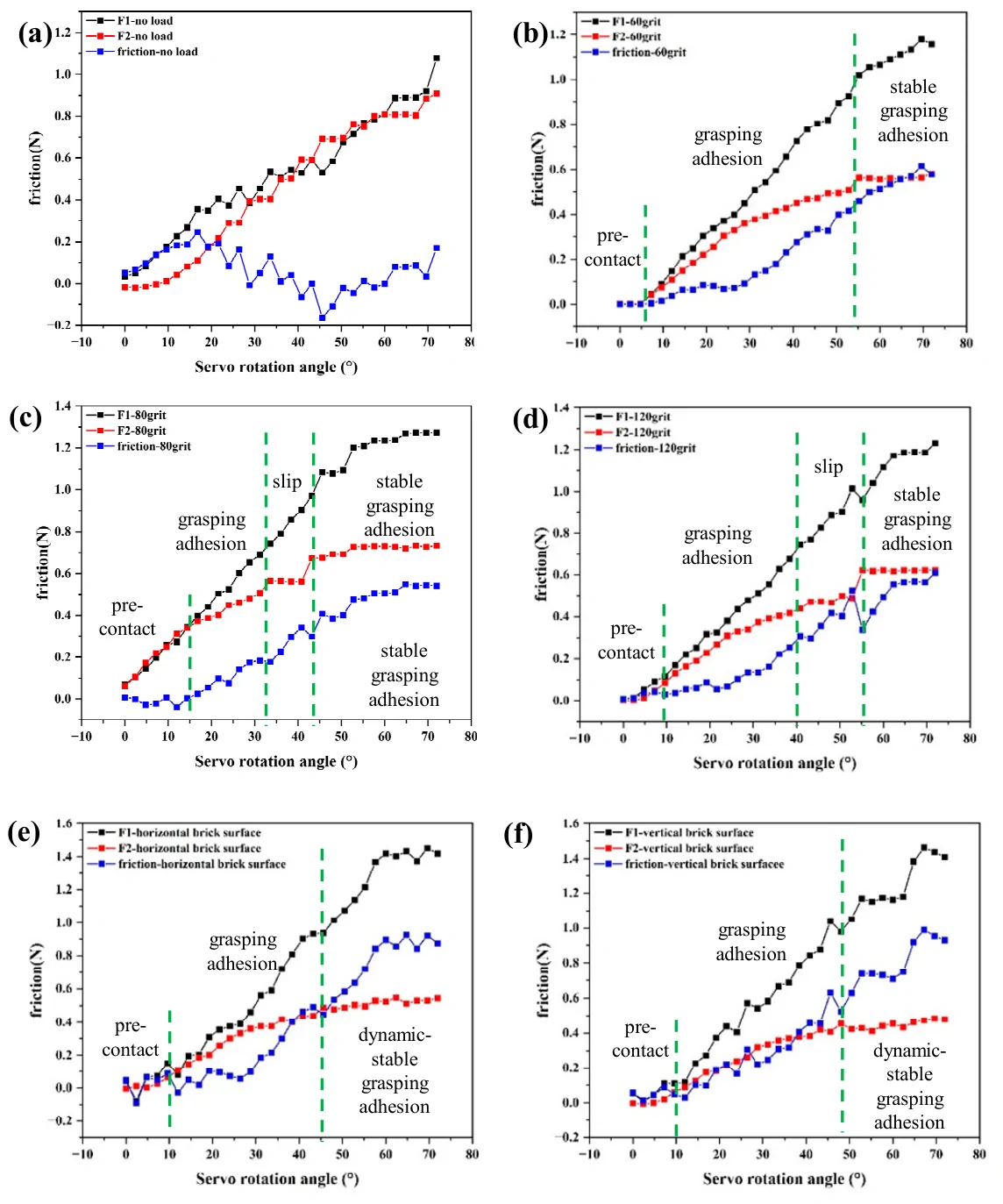
Friction dynamics of a biomimetic flexible spine wall-climbing mechanism on different attachment surfaces: (**a**) Unloaded; (**b**) 60-grit sandpaper; (**c**) 80-grit sandpaper; (**d**) 120-grit sandpaper; (**e**) Horizontal brick surface; (**f**) Vertical brick surface.

The grit number of sandpaper represents the number of abrasive particles per unit area. A higher grit number corresponds to a smaller abrasive-particle diameter, a finer sandpaper surface, and a lower coefficient of friction. The abrasive-particle diameters of 60-grit, 80-grit, and 120-grit sandpaper were 250–297 μm, 177–210 μm, and 125–149 μm, respectively. Because the bilateral opposing claw–spine foot structure was symmetrical and the tangential attachment mechanics of the claw–spine feet on both sides were similar, only the attachment behavior of a single claw–spine foot was analyzed. The frictional mechanical behavior of the bioinspired flexible claw–spine mechanism under different surface conditions is shown in Figure 19.

As shown in Figure 19a, when the bilateral opposing claw–spine feet were suspended without contacting any surface, the extension-spring force, compression-spring force, and tangential claw–spine force gradually increased as the servo-motor angle increased linearly. Under the unloaded condition, the detected tangential attachment force ranged from 0.02 to 0.025 N. This range can be used as a criterion for determining whether the claw–spine structures are in contact with the climbing surface. When the detected value remains within this range for an extended period, the claw–spine structures can be considered not to be in contact with the surface. The main control system can then adjust the posture of the climbing mechanism accordingly, enabling the claw–spine structures to contact the surface and form mechanical interlocking.

As shown in Figure 19b, on the 60-grit sandpaper surface, when the servo-motor angle ranged from 0° to 10°, the claw–spine structures had not yet formed mechanical interlocking with the surface asperities, and the extension-spring force, compression-spring force, and tangential attachment force were close to zero. As the servo-motor angle continued to increase, the claw–spine array gradually formed mechanical interlocking with the surface asperities and entered the stable attachment stage after approximately 60°. The maximum attachment force was 0.62 N. Local force fluctuations during stable attachment were mainly caused by some claw–spine structures disengaging from the original asperities and searching for new stable attachment points.

As shown in Figure 19c, on the 80-grit sandpaper surface, the variations in the mechanical parameters were generally consistent with those observed on the 60-grit sandpaper. When the servo-motor angle ranged from 0° to 19°, the claw–spine structures were in the initial contact stage. From 20° to 45°, the claw–spine array gradually formed mechanical interlocking with the sandpaper asperities. From 45° to 72°, the mechanism entered the stable attachment stage, with a maximum attachment force of 0.54 N. Within the range of 34–43°, the compression-spring force remained temporarily stable, indicating that some claw–spine structures had formed mechanical interlocking, whereas the remaining structures were still sliding and searching for new attachment points.

As shown in Figure 19d, on the 120-grit sandpaper surface, the mechanical parameters generally increased with increasing servo-motor angle, although more sliding stages occurred. As the sandpaper grit number increased, the surface became smoother, making it more difficult for the claw–spine array to form mechanical interlocking with the surface asperities. Consequently, the mechanism underwent multiple sliding stages before entering stable attachment, and the maximum tangential attachment force was 0.61 N.

As shown in Figure 19e, on the horizontal brick surface, the variation trends of the mechanical parameters were generally consistent with those observed on the sandpaper surfaces. Because the surface roughness of the brick was nonuniform, the claw–spine array experienced sliding during attachment before gradually entering a stable attachment state. The maximum attachment force was 0.92 N. When the brick asperities were relatively large, some claw–spine structures formed stable mechanical interlocking, whereas the remaining structures continued sliding over the asperities until the entire claw–spine array achieved stable attachment.

As shown in Figure 19f, on the vertical brick surface, all mechanical parameters increased with increasing servo-motor angle. After undergoing multiple sliding stages, the mechanism entered the stable attachment stage, and the maximum tangential attachment force was 0.99 N. Because the robot’s weight increased the tendency of the claw–spine feet to disengage from the brick surface, the claw–spine array needed to slide continuously and search for attachable asperities until stable mechanical interlocking was formed.

The maximum attachment forces measured on the 60-grit, 80-grit, and 120-grit sandpaper surfaces were 0.62 N, 0.54 N, and 0.61 N, respectively. Among them, the maximum attachment force was highest on the 60-grit sandpaper surface. The maximum attachment force on the 120-grit sandpaper surface was slightly lower than that on the 60-grit surface but higher than that on the 80-grit surface, indicating that the attachment force did not vary monotonically with increasing sandpaper grit.

This phenomenon may be related to the dimensional matching between the surface microstructures of the sandpaper and the claw–spine tips. The abrasive particles on the 60-grit sandpaper were relatively large, and the surface exhibited pronounced asperities and grooves. The claw–spine tips could form strong mechanical interlocking with the larger surface asperities, resulting in a relatively high maximum attachment force. For the 80-grit sandpaper, the abrasive particles were larger than those on the 120-grit sandpaper, and relatively wide and deep gaps, together with large differences in surface height, may have existed between adjacent particles. When the claw–spine array contacted this surface, some claw–spine tips may have fallen into the larger depressions between the particles, thereby reducing the number of effective interlocking contact points and leaving some claw–spine structures suspended or insufficiently engaged. Meanwhile, the small number of claw–spine structures involved in contact may have been subjected to relatively high local loads, leading to local compression, slight sliding, or disengagement. Owing to the uneven load distribution among the claw–spine structures, the array could not fully realize its multipoint coordinated attachment capability, resulting in a relatively low maximum attachment force on the 80-grit sandpaper surface.

In comparison, the abrasive particles on the 120-grit sandpaper were smaller and more densely distributed, while the surface contained a larger number of micro-asperities and exhibited relatively moderate height variations. These densely distributed micro-asperities could provide support and interlocking sites for more claw–spine tips, allowing more claw–spine structures to participate simultaneously in attachment, reducing extensive suspension of the claw–spine structures, and improving the load distribution within the array. In addition, the small gaps between the abrasive particles on the 120-grit sandpaper may have provided better dimensional matching with the claw–spine tips, enabling moderate interlocking and reducing the likelihood of lateral sliding and disengagement. Therefore, the maximum attachment force on the 120-grit sandpaper surface was higher than that on the 80-grit surface.

These results indicate that the attachment performance of the claw–spine array is not determined solely by sandpaper grit or surface roughness. Instead, it is jointly related to the claw–spine tip size, the distribution of surface microstructures, the number of effective interlocking sites, and the load distribution among multiple claw–spine structures. Under the present experimental conditions, the density of microstructures available for claw–spine engagement and their dimensional matching with the claw–spine tips may be important factors affecting the attachment force. This explanation applies only to the claw–spine structure and the 60–120-grit sandpaper surfaces used in this study and cannot be directly extended to higher-grit sandpapers or other types of rough surfaces.

The results may also have been affected by the accuracy of the attachment-force measurement system. In this study, the attachment force was obtained indirectly by measuring the deformation of the springs connected to the claw–spine structures. Therefore, the results were mainly used to compare the attachment processes on different sandpaper surfaces and to provide a reference for the design of the claw–spine attachment-force sensing and adjustment system.

It should be noted that the tests on the horizontal and vertical brick surfaces were mainly conducted to preliminarily verify the attachment feasibility of the designed claw–spine wall-climbing mechanism under different surface orientations, as well as the ability of the measurement system to sense changes in the claw–spine attachment force, rather than to compare the attachment performance under the two surface orientations. Because the distribution of asperity morphology on the brick surface was random, the positions and shapes of the asperities contacted by the claw–spine array varied among tests. Therefore, although the maximum frictional attachment forces measured on the horizontal and vertical brick surfaces were 0.92 N and 0.99 N, respectively, the difference between them was not statistically compared and should not be interpreted as statistically significant. In addition, the current system indirectly senses and adjusts the claw–spine attachment force through the deformation of the springs connected to the claw–spine structures. Consequently, a certain discrepancy remains between the measured results and the actual attachment force generated by the claw–spine structures on rough surfaces, and the corresponding results are mainly used for qualitative analysis and system-function validation.

### 4.3. LSM300 Mechanical Platform Attachment Force Test Results

The tangential attachment friction force and normal attachment force of the claw–spines were measured using the LSM300 force sensor. The acrylic plate placed on the contact surface of the experimental platform was divided into two equal parts. One part was fixed on the horizontal experimental platform, while the other part was connected to the cantilever beam of the force sensor. The two acrylic plates were separated by a 2 mm gap to avoid mutual contact and interference, and both were maintained at the same horizontal level. The joint connecting the two links of the wall-climbing robot was positioned at the center of the gap between the two acrylic plates. The two claw–spine feet of the robot were placed separately on the acrylic plates of the experimental platform and the force sensor, respectively, and were symmetrically distributed about the central axis. Therefore, when the claw–spine feet reached the attachment state, the attachment forces generated by the two feet were theoretically identical, and only one foot was required to be connected to the force sensor for attachment force measurement, as shown in Figure 15c.

**Figure 20:**
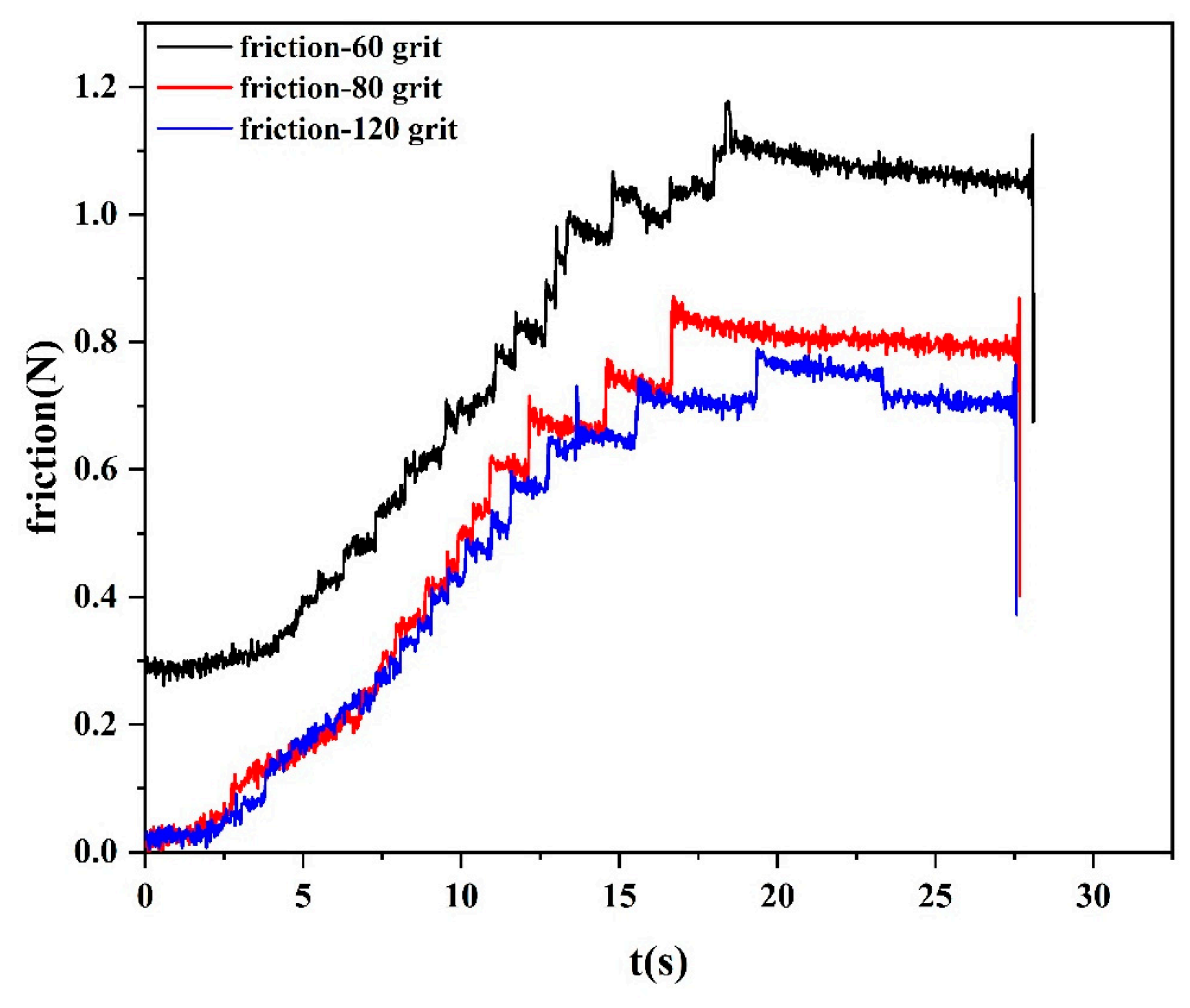
Tangential attachment friction force measurement of the claw–spines using the LSM300 force sensor.

The experimental conditions in this section were identical to those described in Section 4.1. The prototype was placed under the same attachment environment, and the same servo rotation angle was applied to drive the claw–spine foot to adhere to the contact surface. The attachment force generated by the claw–spine foot was measured by the LSM300 force sensor, and the results were compared with those obtained from the previous validation experiments. Sandpapers with different grit sizes were fixed on the acrylic plate connected to the force sensor in Figure 15c. The claw–spine foot performed attachment operations on 60-, 80-, and 120-grit sandpaper surfaces, respectively. The voltage variation in the force sensor during the attachment process was recorded by an oscilloscope. Since the force sensor had been calibrated previously, the voltage variation trend directly represented the variation trend of the claw–spine attachment force. Based on the calibration results, the voltage signals were converted into attachment forces, and the time-dependent variation in the attachment force was obtained, as shown in Figure 20.

Figure 20 shows that the fluctuation stage of the tangential friction force (force step variations) corresponds to the sliding adjustment stage observed in the Hall effect sensor-based detection experiments. This phenomenon occurs because the claw–spine array continuously adjusts its position during the initial attachment process to search for suitable contact locations where mechanical interlocking with surface asperities can be established. Therefore, different degrees of sliding adjustment appeared in all experiments shown in Figure 20. Among them, the claw–spines on the 60-grit sandpaper experienced a shorter adjustment stage and achieved the highest final stable attachment force. In contrast, the 120-grit sandpaper exhibited the longest adjustment stage and resulted in the lowest stable attachment force. These results are consistent with those obtained from the proposed attachment force detection system.

### 4.4. Comparative Analysis of Attachment Friction Force Measurements from the Hall-Effect Sensor and the LSM300

**Figure 21:**
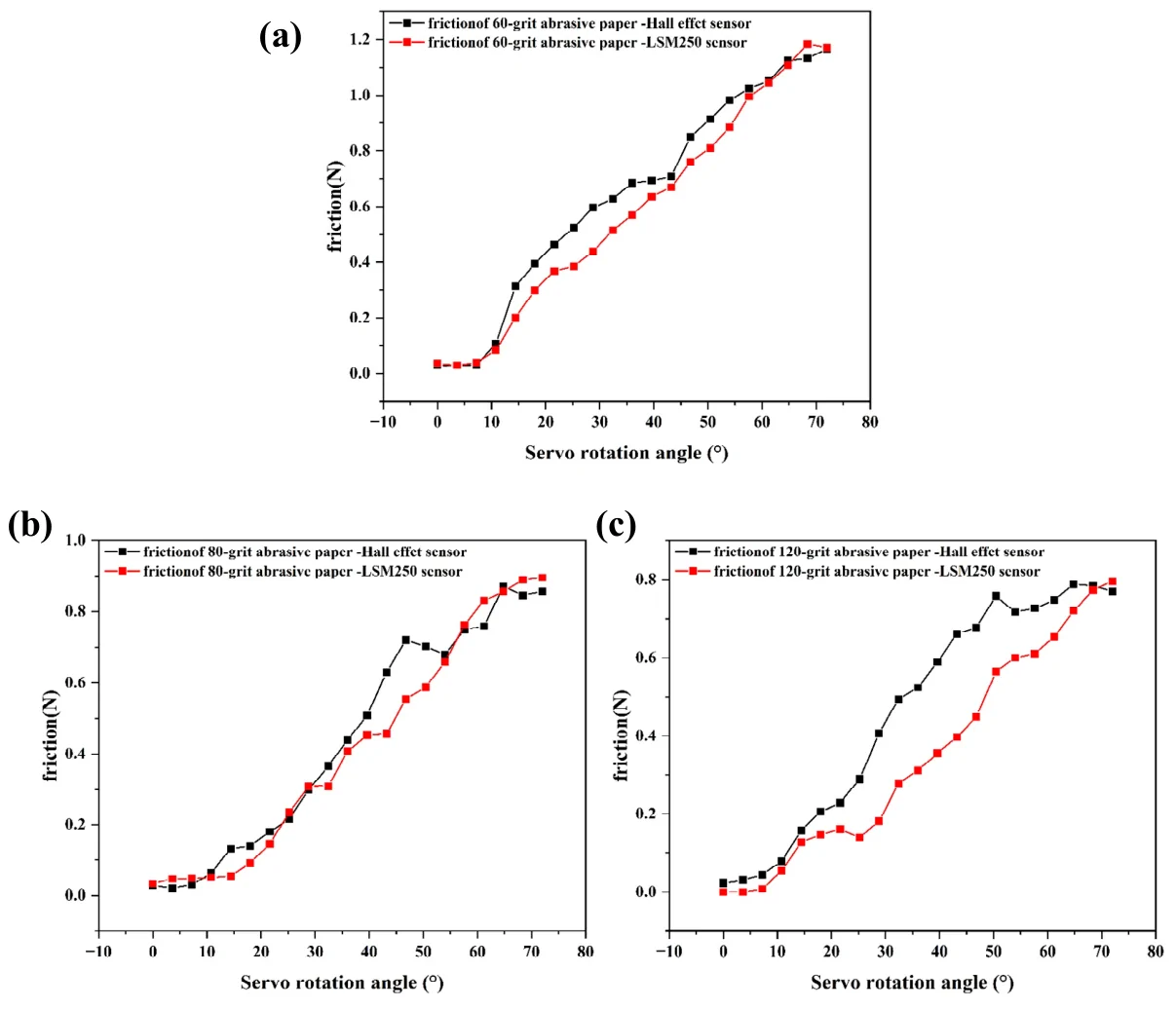
Comparison between the attachment forces detected by the proposed system and measured by the standard force sensor under different surface conditions. (**a**) 60-grit sandpaper; (**b**) 80-grit sandpaper; (**c**) 120-grit sandpaper.

Figure 21a–c show the comparison of attachment friction force measured simultaneously by the Hall-effect sensor-based detection system and the LSM300 sensor when the claw–spine foot adhered to 60-grit, 80-grit, and 120-grit sandpaper surfaces, respectively. When the claw–spine foot adhered to the 60-grit sandpaper, the tangential attachment force reached the maximum value of 1.07 N at approximately 16 s. Subsequently, the attachment friction force remained nearly constant, indicating that the claw–spine foot had reached a stable attachment state. For the 80-grit sandpaper surface, the claw–spine foot reached a maximum attachment force of 0.85 N at approximately 23 s, after which the attachment force remained stable. When adhering to the 120-grit sandpaper surface, the claw–spine foot reached a stable attachment state at approximately 20 s, with a maximum attachment force of 0.78 N. The maximum attachment force obtained on the 120-grit sandpaper was the lowest, whereas that on the 60-grit sandpaper was the highest, which is consistent with the results obtained from the attachment force measurement experiments using the proposed measurement and control system described above. In addition, during attachment tests on the 120-grit sandpaper, the attachment friction force occasionally reached the overall maximum value before the claw–spine foot entered the stable attachment state. This phenomenon was not attributed to a stable attachment condition, but rather to the penetration of the claw–spines into the relatively thin and smooth sandpaper surface, resulting in a temporarily increased resistance force and a false increase in the measured attachment force. Therefore, this transient force peak should be carefully distinguished during attachment state determination to avoid misjudging the stable attachment condition.

During attachment force measurement using the force measurement platform, the stable attachment state of the claw–spine was determined based on the criterion that the tangential attachment force reached its maximum value and remained constant for a certain period. At this stage, the servo motor stopped supplying driving force, and the claw–spine foot maintained a stable attachment state. For the Hall sensor-based attachment force detection system, the determination of the stable attachment state involved two criteria. First, when the compressive spring force no longer changed, the claw–spine array had completed mechanical interlocking with the surface asperities, indicating that the claw–spine foot had entered an initial stable contact state. However, the tangential attachment force continued to increase during this stage. Only when the tangential attachment force reached its maximum value and remained constant was the claw–spine foot considered to have achieved a stable attachment state with the contact surface.

To verify the reliability of the Hall-sensor measurement system, a correlation analysis was conducted between its measurement results and those obtained using the commercial LSM300 sensor on sandpaper surfaces with different grit sizes, as shown in Table 10. The Pearson correlation coefficient and coefficient of determination R^2^ were calculated for the data obtained using the two measurement methods. On the 60- and 80-grit sandpaper surfaces, both the Pearson correlation coefficient and R^2^ were greater than 0.97. On the 120-grit sandpaper surface, both indices remained greater than 0.90. These results indicate a strong positive correlation and a good linear relationship between the measurements obtained using the Hall-sensor system and the LSM300 sensor. The correlation between the two measurement methods was higher on the rougher surfaces, whereas it decreased on the relatively smooth 120-grit sandpaper surface.

The above analysis shows that the Hall-sensor-based attachment-force measurement system and the commercial LSM300 sensor exhibited generally consistent trends in the measured attachment force, preliminarily verifying the feasibility of the proposed measurement system. However, as shown in Figure 21, certain differences remained between the force values measured by the two sensors. It should be noted that the instantaneous maximum force occurring during the attachment process in Figure 21 represents the peak attachment force, rather than the stable attachment force used for the measurement-error calculation. In this study, the stable attachment force was defined as the force value measured after the servo-motor rotation angle reached 72°, when the attachment force entered a relatively stable stage and remained approximately constant for a certain period.

To quantitatively evaluate the consistency between the two measurement methods, the Hall-sensor and LSM300 measurements obtained during the stable attachment stage on the 60-grit, 80-grit, and 120-grit sandpaper surfaces were extracted, and the measurement error was calculated according to Equation (41):

(41) $$\mathrm{Measurement}\;\mathrm{error}\;=\;\frac{\left|F_{\mathrm{Hall}}-F_{\mathrm{LSM}}\right|}{F_{\mathrm{Hall}}}\;\times \;100\%$$

As shown in Table 11, the measurement errors between the two methods for the stable attachment forces on the 60-grit, 80-grit, and 120-grit sandpaper surfaces were 0.43%, 4.41%, and 3.27%, respectively. The average measurement error was 2.70%, and the maximum error was 4.41%. All three measurement errors were within 10%, indicating that the attachment-force measurements obtained using the Hall-sensor-based system were in good agreement with those obtained using the commercial LSM300 sensor during the stable attachment stage. These results verify the feasibility of the proposed attachment-force measurement system for sensing changes in the claw–spine attachment force. It should be noted that the system indirectly determines the attachment force by measuring the deformation of the springs connected to the claw–spine structures; therefore, a certain difference may still exist between the measured force and the actual force acting at the claw–spine tips.

## 5. Conclusions

In this study, the attachment friction mechanism between claw–spines and rough surfaces was investigated to reveal the bio-inspired mechanism of flexible claw–spines. A mechanical model for the attachment force of a single claw–spine on arbitrary surfaces was established. Based on the theoretical analysis, a claw–spine attachment force detection method was developed, and a bio-inspired climbing mechanism together with its hardware and software measurement and control platforms was fabricated. Finally, comparative experiments were conducted to verify the feasibility and accuracy of the proposed detection system. The main conclusions are summarized as follows:

(1) A stiffness-matrix-based frictional mechanics model for claw–spine attachment on surfaces of arbitrary geometry, together with a bilateral opposing attachment model for claw–spine structures in contact with the surface, was proposed. The force states of the claw–spine structures during contact and attachment to arbitrary curved surfaces were analyzed, the mechanical expressions for the claw–spine attachment force were obtained, and the criteria for stable attachment and the corresponding safe range of frame displacement were derived.
(2) Based on the directional attachment mechanics model of a single claw–spine structure on rough surfaces, a bilateral opposing attachment structure was designed. The attachment-force sensing principle and attachment-force measurement system were investigated, including the measurement principle of the system. A bilateral opposing claw–spine foot attachment system was developed, and the models of the key modules used in the measurement scheme were selected, thereby establishing the hardware basis for attachment-force measurement and control.
(3) Quasi-static attachment tests under different surface conditions showed that the maximum attachment forces of the bioinspired claw–spine mechanism on 60-grit, 80-grit, and 120-grit sandpaper surfaces were 0.62, 0.54, and 0.61 N, respectively. The maximum attachment forces on horizontal and vertical brick surfaces were 0.92 and 0.99 N, respectively.
(4) During the experiments, the developed attachment-force measurement system was used to measure the quasi-static attachment-force variations in the claw–spine foot on various rough surfaces, and the results were compared with those obtained using a commercial sensor (LSM300). By comparing the measured and detected values of the stable frictional attachment force and calculating the weighted average of the measurement errors under different environmental conditions, the attachment-force measurement error of the claw–spine system was determined to be 10%. The results demonstrate that the friction mechanics behavior at the claw–spine attachment interface can be used to detect variations in the attachment force state during robot locomotion, thereby validating the relevant theoretical analysis and confirming the reliability and accuracy of the proposed system. The system enables quasi-static tests during robot locomotion by slowly increasing the servo motor rotation angle, allowing the detection of the claw–spine attachment force. This demonstrates the practicality of the proposed system and provides key technical support for the subsequent development of wall-climbing robot systems.
(5) This study investigated the mechanical behavior between the designed claw–spine structure and rough contact surfaces, and subsequently developed a system with claw–spine attachment force sensing and perception capabilities. The proposed system indirectly evaluates the attachment state by measuring the deformation of the spring connected to the claw–spine structure, rather than directly measuring the attachment force between the claw–spine tip and the contact surface. Therefore, direct measurement of the contact force between the claw–spine tip and the contact surface has not yet been achieved. Furthermore, the proposed method is mainly applicable to attachment measurements on relatively rough surfaces. For relatively smooth surfaces, such as 120-grit sandpaper, the measurement reliability decreases. The measured results still exhibit certain differences from the actual attachment force generated by the claw–spine on rough wall surfaces, and the current system can only achieve qualitative characterization of the attachment state. In addition, the effects of contaminants and durability issues have not yet been systematically investigated through experiments. The results of this study can provide theoretical references for the design of claw–spine attachment force sensing and regulation systems.

## Figures and Tables

**Table 1:** Material Properties of Nylon and Acupuncture Needles.

Material	PA2200 (ESO)	Stainless Steel
$\mathrm{Density}\;({g/cm}^{3})$	0.93	7.8
Elongation	20 ± 5%	/
Young’s modulus (MPa)	1700	200,000
Poisson’s ratio	0.35	0.3
Tensile strength (MPa)	45 ± 3	250

**Table 2:** Properties of Photopolymer Resins.

Property	Parameters
Viscosity	340 cps (30 °C)
Density	$1.16\;g/{cm}^{3}$
Flexural Strength	47.2~47.6 MPa
Tensile Strength	33.8~40.2 MPa
Elongation at Break	6~9%
Elongation in Bending	3%
Modulus of Elasticity	2370~2650 MPa
Poisson’s Ratio	0.41
Flexural Strength	66.8~67.8 MPa
Bending Coefficient	2178~2222 MPa
Impact Strength	23~29 J/cm
High-Speed Puncture Strength	4.60 J
Hardness	79
Water Absorption	0.4%

**Table 3:** Calibration of Hall Sensor Displacement Measurements.

Specified Distance (mm)	Actual Measured Distance (mm)	Specified Distance (mm)	Actual Measured Distance (mm)
0.02	0.019	0.06	0.057
0.03	0.029	0.07	0.063
0.04	0.038	0.08	0.078
0.05	0.049	0.09	0.085

**Table 4:** Data acquired using the test platform.

Displacement (mm)	Output Voltage (V)	Displacement (mm)	Output Voltage (V)
1	0.550	11	1.575
2	0.868	12	1.610
3	1.084	13	1.655
4	1.226	14	1.712
5	1.324	15	1.791
6	1.392	16	1.901
7	1.444	17	2.062
8	1.481	18	2.308
9	1.514	19	2.751
10	1.544	20	2.994

**Table 5:** Data Acquisition and Processing for the Verification Mechanism of the Detection Principle.

Servo Angle (°)	Spring Force (N)	Servo Angle (°)	Spring Force (N)
0	0.00724	32.4	0.18145
3.6	0.00943	36	0.22657
7.2	0.01254	39.6	0.31944
10.8	0.02196	43.2	0.29933
14.4	0.03468	46.8	0.39676
18	0.03961	50.4	0.40208
21.6	0.09932	54	0.47776
25.2	0.10891	57.6	0.50045
28.8	0.17496	61.2	0.54866

**Table 6:** Material parameters of each component in a single bio-inspired flexible claw–spine·.

Component	Material	Young’s Modulus/MPa	Poisson’s Ratio	Mesh Size/mm
Flexible Connector	VYtaFleX20 Polyurethane Rubber	0.345	0.49	0.50
Rigid Connector	PA2200 Nylon	1700	0.35	0.20
Rigid Spine	Stainless Steel	200,000	0.31	0.05
Bonding Layer	3M DP100 Plus Clear Epoxy Adhesive	10.413	0.39	0.10

**Table 7:** Configuration of Different Displacement and Operating Conditions.

Deformation Level	Operating Condition	Displacement in the X-Direction (mm)	Displacement in the Y-Direction (mm)	Angle of Rotation About the Z-Axis (°)
0.5	a1	−0.5	0	0
0.5	a2	0	−0.5	0
0.5	a3	0	0	−0.5
1.0	b1	−1.0	0	0
1.0	b2	0	−1.0	0
1.0	b3	0	0	−1.0
1.5	c1	−1.5	0	0
1.5	c2	0	−1.5	0
1.5	c3	0	0	−1.5

**Table 8:** Maximum Total Deformation, Equivalent Elastic Strain, and Equivalent Stress for Each Operating Condition.

Operating Condition	Loading Method	Maximum Total Deformation (mm)	Maximum Equivalent Elastic Strain (mm)	Maximum Equivalent Stress (MPa)
a1	0.5 mm displacement in the X-direction	0.50773	0.015481	0.070632
a2	0.5 mm displacement in the Y-direction	0.50021	0.0041336	0.023287
a3	0.5 mm displacement in the Y-direction	0.054469	0.0016973	0.025317
b1	Displacement in the X-direction by 1.0 mm	1.0156	0.030861	0.13764
b2	Displacement in the Y-axis by 1.0 mm	1.0005	0.0078047	0.044313
b3	Rotation around the Z-axis by 1.0°	0.10894	0.0033997	0.017174
c1	Displacement in the X-axis by 1.5 mm	1.5237	0.046116	0.20112
c2	Y-axis displacement 1.5 mm	1.5008	0.011014	0.063351
c3	Rotation around the Z-axis 1.5°	0.16341	0.0051066	0.025896

**Table 9:** Results of Reaction Forces and Reaction Torques for Various Operating Conditions.

Operating Condition	F_X_/N	F_Y_/N	M/N·mm
a1	−3.047 $\times \;10^{-4}$	−1.712 $\times \;10^{-5}$	1.122 $\times \;10^{-3}$
a2	−8.935 $\times \;10^{-7}$	−1.601 $\times \;10^{-6}$	3.096 $\times \;10^{-4}$
a3	2.015 ${\times \;10}^{-5}$	5.752 $\times \;10^{-6}$	−1.243 $\times \;10^{-4}$
b1	−6.045 $\times \;10^{-4}$	−3.212 $\times \;10^{-5}$	2.193 $\times \;10^{-3}$
b2	−1.555 $\times \;10^{-5}$	−6.221 ${\times \;10}^{-5}$	5.828 $\times \;10^{-4}$
b3	5.885 ${\times \;10}^{-5}$	4.542 ${\times \;10}^{-5}$	−2.502 $\times \;10^{-4}$
c1	−8.996 $\times 10^{-4}$	−4.501 ${\times \;10}^{-5}$	3.215 $\times \;10^{-3}$
c2	−8.096 $\times \;10^{-6}$	−9.370 $\times \;10^{-5}$	8.232 $\times \;10^{-4}$
c3	6.112 $\times \;10^{-5}$	1.737 ${\times \;10}^{-5}$	−3.773 $\times 10^{-4}$

**Table 10:** Correlation analysis of the measurement results obtained using the Hall sensor and LSM300 on sandpaper surfaces with different grit sizes.

Sandpaper Grit (Grit)	Pearson Correlation Coefficient (r)	Coefficient of Determination (R^2^)
60	0.9887	0.9775
80	0.9787	0.9787
120	0.9494	0.9014

**Table 11:** Measurement Errors between the Hall-Sensor-Based Attachment-Force Measurement System and the LSM300 Sensor on Sandpaper Surfaces with Different Grit Sizes at a Servo-Motor Angle of 72°.

Sandpaper Grit (Grit)	Hall-Sensor Measurement (N)	LSM Measurement (N)	Measurement Error (%)
60	1.1671	1.1721	0.43
80	0.8572	0.8950	4.41
120	0.7703	0.7955	3.27

## Data Availability

The original contributions presented in the study are included in the article.
